# Real-Time Fluorescence Measurements of ROS and [Ca^2+^] in Ischemic / Reperfused Rat Hearts: Detectable Increases Occur only after Mitochondrial Pore Opening and Are Attenuated by Ischemic Preconditioning

**DOI:** 10.1371/journal.pone.0167300

**Published:** 2016-12-01

**Authors:** Tatyana Andrienko, Philippe Pasdois, Andreas Rossbach, Andrew P Halestrap

**Affiliations:** 1 School of Biochemistry and Bristol Cardiovascular, Biomedical Sciences Building, University of Bristol, Bristol, United Kingdom; 2 INSERM U1045—L'Institut de Rythmologie et Modélisation Cardiaque (LIRYC), Université de Bordeaux, Bordeaux, France; Emory University, UNITED STATES

## Abstract

Mitochondrial permeability transition pore (mPTP) opening is critical for ischemia / reperfusion (I/R) injury and is associated with increased [Ca^2+^] and reactive oxygen species (ROS). Here we employ surface fluorescence to establish the temporal sequence of these events in beating perfused hearts subject to global I/R. A bespoke fluorimeter was used to synchronously monitor surface fluorescence and reflectance of Langendorff-perfused rat hearts at multiple wavelengths, with simultaneous measurements of hemodynamic function. Potential interference by motion artefacts and internal filtering was assessed and minimised. Re-oxidation of NAD(P)H and flavoproteins on reperfusion (detected using autofluorescence) was rapid (t_0.5_ < 15 s) and significantly slower following ischemic preconditioning (IP). This argues against superoxide production from reduced Complex 1 being a critical mediator of initial mPTP opening during early reperfusion. Furthermore, MitoPY1 (a mitochondria-targeted H_2_O_2_-sensitive fluorescent probe) and aconitase activity measurements failed to detect matrix ROS increases during early reperfusion. However, two different fluorescent cytosolic ROS probes did detect ROS increases after 2–3 min of reperfusion, which was shown to be after initiation of mPTP opening. Cyclosporin A (CsA) and IP attenuated these responses and reduced infarct size. [Ca^2+^]_i_ (monitored with Indo-1) increased progressively during ischemia, but dropped rapidly within 90 s of reperfusion when total mitochondrial [Ca^2+^] was shown to be increased. These early changes in [Ca^2+^] were not attenuated by IP, but substantial [Ca^2+^] increases were observed after 2–3 min reperfusion and these were prevented by both IP and CsA. Our data suggest that the major increases in ROS and [Ca^2+^] detected later in reperfusion are secondary to mPTP opening. If earlier IP-sensitive changes occur that might trigger initial mPTP opening they are below our limit of detection. Rather, we suggest that IP may inhibit initial mPTP opening by alternative mechanisms such as prevention of hexokinase 2 dissociation from mitochondria during ischemia.

## Introduction

Reperfusion of the heart following prolonged ischemia causes irreversible damage through myocyte death and resulting infarct formation. Critical to this process is opening of the mitochondrial permeability transition pore (mPTP) that occurs after about 2 min of reperfusion, when the intracellular pH (pHi) returns to preischemic values from the low ischemic values (<6.5) that inhibit mPTP opening [1,2]. MPTP opening compromises cellular bioenergetics, impairing restoration of ionic homeostasis, including [Ca^2+^], while also increasing reactive oxygen species (ROS) production. These effects together produce further mPTP opening and bioenergetic compromise leading to a spreading wave of necrotic cell death that constitutes the infarct [3,4]. Cardioprotection is afforded by pharmacological inhibition of mPTP opening by Cyclosporin A (CsA) [5], Sanglifehrin A [6] or cinnamic anilides [7], as well as by regimes such as ischemic preconditioning (IP) and post-conditioning which also act, at least in part, by preventing mPTP opening [3,4].

Although the exact molecular composition of the mPTP remains uncertain [8–10], it is well established that its opening is triggered by elevated matrix [Ca^2+^], while the sensitivity to [Ca^2+^] is greatly increased by oxidative stress, elevated phosphate and decreased matrix adenine nucleotides [3]. These are all conditions associated with ischemia / reperfusion (I/R), and we, like many others, have proposed that the main triggers for mPTP opening during early reperfusion are an increased matrix [Ca^2+^] together with mitochondrial ROS production [3,8]. Furthermore, it has been proposed that IP reduces or prevents mPTP opening, and thus I/R injury, by attenuating these triggers [3,11]. While evidence in support of this view has come from studies on isolated cardiac myocytes subject to simulated I/R [12,13], we were concerned that the conditions experienced by isolated myocytes subject to simulated I/R do not adequately reproduce those occurring in the intact heart subject to I/R. In particular, the beating perfused heart exhibits much higher metabolic turnover and calcium cycling rates than isolated cardiac myoctes which also cannot mimic the complex cell / cell interactions occurring in the whole heart. Hence we wished to monitor ROS and [Ca^2+^] dynamics in the perfused beating heart subject to I/R.

Others have monitored dihydroethidium (DHE) surface fluorescence of the perfused heart to detect ROS production during I/R [14,15]. However, major concerns have been expressed over the use of DHE as a ROS probe, both in terms of whether the fluorescent species monitored really detects superoxide [16] and its sensitivity to changes in mitochondrial membrane potential [17]. The luminescent probe lucigenin has also been employed which did detect a large increase in ROS during reperfusion, but this only reached a peak after about 5 min of reperfusion [17] which is after mPTP opening [1,2]. Others have used multi-photon microscopy to measure changes of [Ca^2+^] and ROS in the perfused heart [18], but in these studies pharmacological inhibition of contractile function was required. This would have greatly reduced ATP turnover and thus the energetic demand on mitochondria which may well have caused substantial modulation of the kinetics of mPTP opening during reperfusion when compared to the beating heart. Although sophisticated gating mechanisms can be employed in the beating heart to correct for motion artefacts [19], such an approach would only report changes in a few cardiomyoctes and thus might not reflect the behaviour of the majority. Others have used mass spectrometry probes or protein carbonylation to detect increases in mitochondrial superoxide and other ROS species during reperfusion [11,20]. However, these techniques required the hearts to be freeze-clamped at a specific time and, unlike continuous fluorescent measurements, do not have the time resolution to detect whether this ROS increase precedes or follows mPTP opening.

Here, we report data from experiments in which we continuously monitored surface fluorescence of the beating heart at multiple excitation and emission wavelengths using a bespoke fluorimeter that also monitors surface reflectance. We have measured endogenous NAD(P)H and flavoprotein fluorescence as well as intracellular [Ca^2+^] with Indo-1 and ROS with both cytosolic and mitochondria-targeted ROS probes. In parallel we measured aconitase activity and total [Ca^2+^] in mitochondria isolated after 90 s reperfusion as additional indicators of mitochondrial ROS and [Ca^2+^] changes. Contrary to our expectations, we were unable to detect a significant increases in ROS until after about 1.5–2 min of reperfusion, which was after mPTP opening had occurred. Furthermore, the progressive increase in ROS seen after this time point in reperfusion was attenuated by inhibiting mPTP opening with Cyclosporin A (CsA) or IP, both of which reduced infarct size. Measurement of [Ca^2+^]_i_ with Indo-1 showed it to increase progressively during ischemia, and drop rapidly within 90 s of reperfusion when total mitochondrial [Ca^2+^] was increased. While these early changes in [Ca^2+^] were not attenuated by IP, a substantial [Ca^2+^] increase was observed after 2–3 min reperfusion that was prevented by both IP and CsA and thus likely to be secondary to mPTP opening. We conclude that any early IP-sensitive changes in [Ca^2+^] and ROS that might trigger initial mPTP opening are too small to be detected. Rather, we propose that IP may inhibit initial mPTP opening by alternative mechanisms such as prevention of hexokinase 2 dissociation from mitochondria during ischemia [21,22]. Subsequent increases in ROS and [Ca^2+^] follow this initial mPTP opening and so are also attenuated by IP. This may prevent a progressive cycle of mPTP opening, ROS production and calcium overload that would lead to infarct formation.

## Materials and Methods

### Materials

Unless otherwise stated chemicals and reagents used in this study were purchased from Sigma-Aldrich (St. Louis, MO) or Fisher Scientific UK. 5-carboxy-2’,7’-dichlorofluoroscein diacetate, di(acetoxymethyl ester) was from Anaspec (Fremont, CA), but is no longer commercially available. Calcein-AM was from Biotium (Hayward, CA), MitoPY1 and PO1 from Tocris Bioscience (R & D Systems Europe LTD, Abingdon, UK) and Indo-1-AM from TEFLabs (Austin, TX). Invitrogen/Molecular Probes (Eugene, OR) supplied Pluronic F-127, Fura-FF—pentapotassium salt, dihydroethidium, 2',7'-dichlorodihydrofluorescein diacetate, 6-carboxy-2',7'-dichlorodihydrofluorescein diacetate, 6-carboxy-2',7'-dichlorodihydrofluorescein diacetate, di(acetoxymethyl ester) and MitoTracker Deep Red FM.

### Methods

#### Langendorff heart perfusion

**Fig 1:**
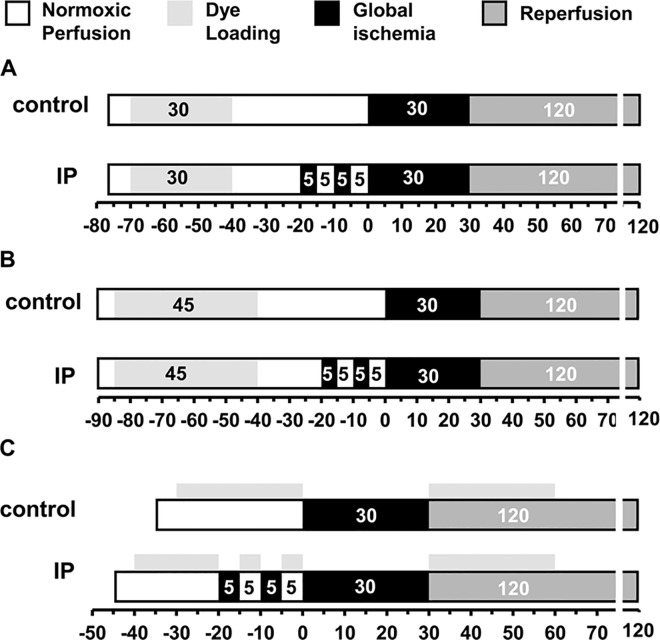
Perfusion protocols used for fluorescence measurements. **A,** A 30 min dye loading protocol was used for loading the heart with 5 μmol/L 5-cH_2_DCFDA, diAM, 0.4 μmol/L calcein-AM or 3 μmol/L MitoPY1. **B,** A 45 min dye loading protocol with recirculation was used to load the heart with 3 μmol/L Indo-1. **C,** Hearts were perfused with 5 μmol/L PO1 for 30 min before index ischemia and 30 min on reperfusion. Further details are given in Supporting Information.

All procedures conformed to the UK Animals (Scientific Procedures) Act 1986 and the guidelines from the Directive 2010/63/EU of the European Parliament and were approved by the local ethical committees of the University of Bristol (UB/09/012) and University of Bordeaux (Authorization number A33-318-2). Hearts from male Wistar rats (250–300 g) were perfused with Krebs–Henseleit Buffer (KHB) in Langendorff mode at constant flow (12 mL/min) with hemodynamic function determined using a water-filled latex balloon inserted into the left ventricle essentially as described previously [23]. IP was elicited by two cycles of 5 min of global ischemia prior to 30 min global (index) ischemia as summarised schematically in Fig 1. After 120 min reperfusion hearts were analysed for infarct size using 2’,3’,5’-triphenyltetrazolium chloride (TTC) staining [21]. A more extensive description of the methods used of the techniques used for heart perfusion is provided in Supporing Information. Measurement of heart succinate content was performed by freeze-clamping the heart followed by acidic extraction of metabolites as described previously [21] and enzymatic assay of succinate [24].

#### Whole heart surface fluorescence measurements

**Fig 2:**
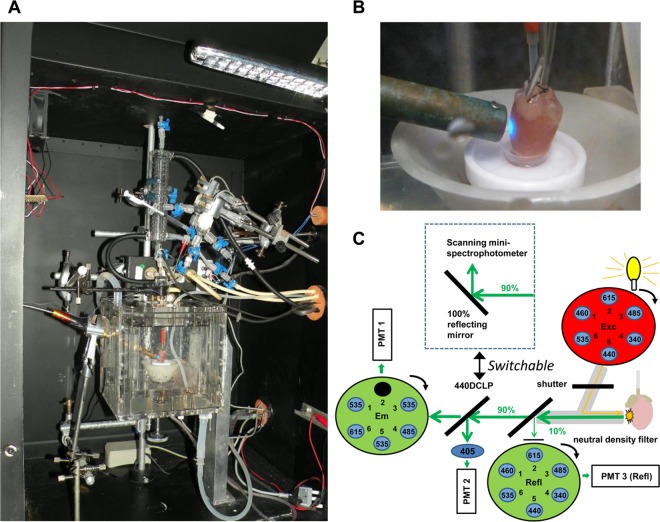
Design of the bespoke surface fluorescence apparatus. **A,** Picture of the interior of the light-proof box showing the water jacketed perfusion chamber containing a Langendorff-perfused rat heart. **B,** Zoom in of the perfusion chamber to show that the light is shining on the surface of the left ventricular free wall and that motion artefact was minimized by placing hearts’ apex in a small retaining cup. **C,** Schematic of the optics. Insert shows alternative configuration for measuring surface fluorescence spectra by switching between the 440 DCLP dichroic mirror for filter based measurements and a 100% reflecting mirror for fluorescence spectrum measurements.

Epicardial fluorescence was monitored using a spinning-wheel fluorimeter designed and custom-built by the authors (PP and APH) in collaboration with Cairn Research Ltd, Faversham, Kent, ME13 8UP. For these experiments, a modified perfusion apparatus was employed that was contained within a light-proof box accommodating the optic fiber from the fluorimeter. This optic fibre was placed at 2–3 mm distance from the left ventricular wall of the heart which was maintained at 37°C in a water jacketed and humidified Plexiglas perfusion chamber throughout the perfusion. The equipment is shown in Fig 2A and 2B and illustrated schematically in Fig 2C. Details of the filters used are given in S1 Table which also includes photomultiplier voltage settings. A more extensive description of the equipment and techniques used for surface fluorescence measurements is provided in Supporting Information.

#### Autofluorescence of NAD(P)H and flavoproteins

These were measured using excitation at 340 and 460 nm and emission 485 and 535 nm, respectively. For measurements of the rapid changes of autofluorescence seen on reperfusion data were collected continuously at 10 Hz for the last 15 s of ischemia and the first 2 min of reperfusion.

#### ROS and [Ca^2+^] measurements

The fluorescent dyes used to monitor ROS were 5-carboxy-2’,7’-dichlorodihydrofluorescein diacetate, di(acetoxymethyl ester) (5-cH_2_DCFDA, diAM Ex 485 nm, Em 535 nm), Peroxy-Orange 1 (PO1, Ex 535 nm, Em 615 nm) and Mitochondria Peroxy-Yellow 1 (MitoPY1, Ex 485 nm, Em 535 nm) while Indo-1-AM (Ex 340 nm Em 405 nm and 485 nm) was used to monitor [Ca^2+^]. Calcein-AM (Ex 485 nm, Em 535 nm) was employed as a negative control. The protocols used to load these dyes are shown in Fig 1 and it should be noted that for the protocols shown in Fig 1A and 1B, 10 nmol/L insulin was present in the KHB perfusion medium during dye loading to maintain glycogen levels over the extended pre-ischemic perfusion period. In its absence glycogen became depleted and this gave significant cardioprotection [21,25]. For PO1 the dye was not pre-loaded, but present throughout the perfusion (Fig 1C) and insulin was not required. In all experiments employing probes loaded in their acetoxymethyl ester (AM) form, the KHB was also supplemented with 0.1 mmol/L probenecid to limit dye leakage while Pluronic F-127 was added to the stock solutions of the dyes to aid their solubilisation. Dye loading started about 7–8 min after the heart cannulation and was preceded by measurement of background fluorescence for 3–5 min.

#### Cardiomyocyte isolation and confocal imaging of MitoPy1

Rat ventricular myocytes were isolated from male Wistar Rats (250g) with a modified Langendorff method as described previously [26]. Cells were immobilised on laminin-coated round glass coverslips, cultured in 6-well plates in medium M199 and used immediately following isolation or after an overnight incubation. Myocytes were washed into HEPES-bufffered saline solution containing (in mmol/L): NaCl 130, HEPES 25, KCl 5, glucose 10, MgCl_2_ 1, and CaCl_2_ 1.8 and loaded with MitoTracker Deep Red (200 nmol/L) in the presence or absence of 14 μmol/L MitoPY1 for 30 min at 37°C. Confocal microscopy was carried out on a Leica SP-5 microscope (63.0 x1.40 Oil) at 37°C in the Woolfson Bio-imaging Centre at the University of Bristol (http://www.bristol.ac.uk/wolfson-bioimaging/). After loading, 2.5 ml HEPES-bufffered saline solution was added to the cells. H₂O₂ was added directly to the coverslips (final concentration 100 μmol/L), incubated for 10 minutes and the cells were imaged again. MitoPY1 was excited by Argon laser (514 nm line, 100%) and its fluorescence was collected at 520–570 nm. MitoTracker Deep Red was excited by HeNe laser (633 nm line, 4.1%) and its fluorescence was collected at 640–700 nm.

#### Isolation of heart mitochondria

Mitochondria were isolated from rat hearts as described previously [21] using Dounce-Potter homogenisation of protease-treated tissue in isolation medium (300 mmol/L sucrose, 10 mmol/L Tris, 2 mmol/L EGTA pH 7.4 at 4°C), followed by differential centrifugation and Percoll (25%) gradient centrifugation.

#### Measurement of ROS production and mPTP opening in isolated mitochondria

Opening of the mPTP was followed at 25°C in energised mitochondria as described previously [27] using fluorescence measurements of extramitochondrial [Ca^2+^] (Fura-FF) and ΔΨ (Rhodamine-123) together with light scattering (LS) at 560 nm monitored continuously with a Cairn Research spinning-wheel multiwavelength fluorimeter. The buffer (pH 7.2) contained (in mmol/L): KCl 125, MOPS 20, Tris 10, KH_2_PO_4_ 2.5 plus 10 μmol/L EGTA, 1 μmol/L Fura-FF pentapotassium salt, 100 nmol/L Rhodamine-123 and respiratory substrates (5 mmol/L L-glutamate + 2 mmol/L L-malate + 5 mmol/L succinate (GMS)). H_2_O_2_ production was measured immediately afterwards in the same apparatus using Amplex Red (10 μmol/L) and peroxidase (0.1 mg/mL) as described previously [28] but with simultaneous monitoring of LS.

#### Measurement of mitochondrial enzymes activities and total calcium content

Mitochondria were isolated from rat hearts as described above using a modified isolation buffer lacking EGTA but enriched with 0.2 μmol/L of Cyclosporin A, 0.5 μmol/L of ruthenium red and 2 mg/mL of free fatty acid BSA. At the end of the perfusion protocol (S1 Fig), the heart was flushed with 12 mL of modified isolation buffer at 4°C prior to mitochondrial isolation. Mitochondria were divided into aliquots and immediately frozen in liquid nitrogen for further biochemical measurements. Citrate synthase activity was assessed as previously described [21] while aconitase activity was determined at 37°C using a modification of that described by Chouchani *et al*. [20]. The assay buffer used was: KH_2_PO_4_ 50 mmol/L; Triton X-100 0.1% (w/v); NADP^+^ 0.2 mmol/L; MnCl_2_ 0.6 mmol/L, pH 7.4 with KOH. Purified mitochondria were incubated in 1 mL of the assay buffer supplemented with 2 units/mL of NADP-dependent isocitrate dehydrogenase (Leebio products). The reaction was started by the addition of 5 mmol/L of sodium-citrate (pH 7.4) and the production of NADPH followed at 340 nm. In the absence of citrate no NADPH production was detected. To evaluate aconitase sensitivity to H_2_O_2_ in the different groups mitochondria were incubated for 5 min on ice in the presence of 200 μmol/L of H_2_O_2_. The assay was then performed as described above (final H_2_O_2_ concentration in the cuvette 20 μmol/L).

For determination of total calcium content, 200 μg of the mitochondria were mixed with an equal volume of 0.6 mol/L HCl. The mixture was then sonicated two times for 10 s at full power using a Fisherbrand FB15062 sonicator interspersed with a 10 s rest interval. The final homogenate was used to evaluate mitochondrial total calcium content (expressed as ngatoms/mg protein) using the α-cresolphthalein complexone assay (Cayman Chemical), according to the manufacturer’s instructions. Preliminary experiments were performed to confirm that 15 μL of a mixture containing an equal volume of modified ISA and 0.6 mol/L HCl did not alter the pH of the assay buffer (210 μL final volume in the well).

#### Measurement of mPTP opening in hearts using mitochondrial calcein retention

After the stabilization period, Langendorff-perfused hearts were loaded for 10 min with 0.4 μmol/L final of calcein-AM (see S1 Fig). Purified mitochondria (300 to 500 μg) were resuspended in 2 ml final of assay buffer (KH_2_PO_4_ 33 mmol/L, Triton X-100 0.1% (w/v), pH 7.2) in order to determine the calcein emission spectra using a Xenius fluorimeter (Safas, Monaco) with the following settings: PMT = 900 volts, bandwidth = 10 nm, Ex 493 nm, Em scanned from 505 to 540 nm with a 0.2 nm increment. The maximum emission obtained in the different populations of mitochondria were calibrated using a standard curve obtained using known quantities of calcein. Mitochondrial calcein content was expressed as nmol per mg protein. Background fluorescence was determined in parallel experiments with mitochondria isolated from hearts (n = 3) subjected to a mock loading (DMSO vehicle only).

### Statistical analysis

Data are presented as means ± SEM for the number of separate heart perfusions or mitochondrial preparations indicated. Statistical analysis was performed using the relevant functions in Excel, GraphPad Prism 6 or SPSS 17.0. For real-time fluorescence traces, a 2-tailed *t*-test was employed to determine the significance of differences between control and IP or drug-treated hearts. An F-test was used first to check the variances and if unequal a heteroscedastic *t*-test was used. For analysis of differences in infarct size and hemodynamic function between groups a 1-way ANOVA followed by Tukey's multiple comparisons test was used.

## Results

### Validation of the surface fluorescence measurements

**Fig 3:**
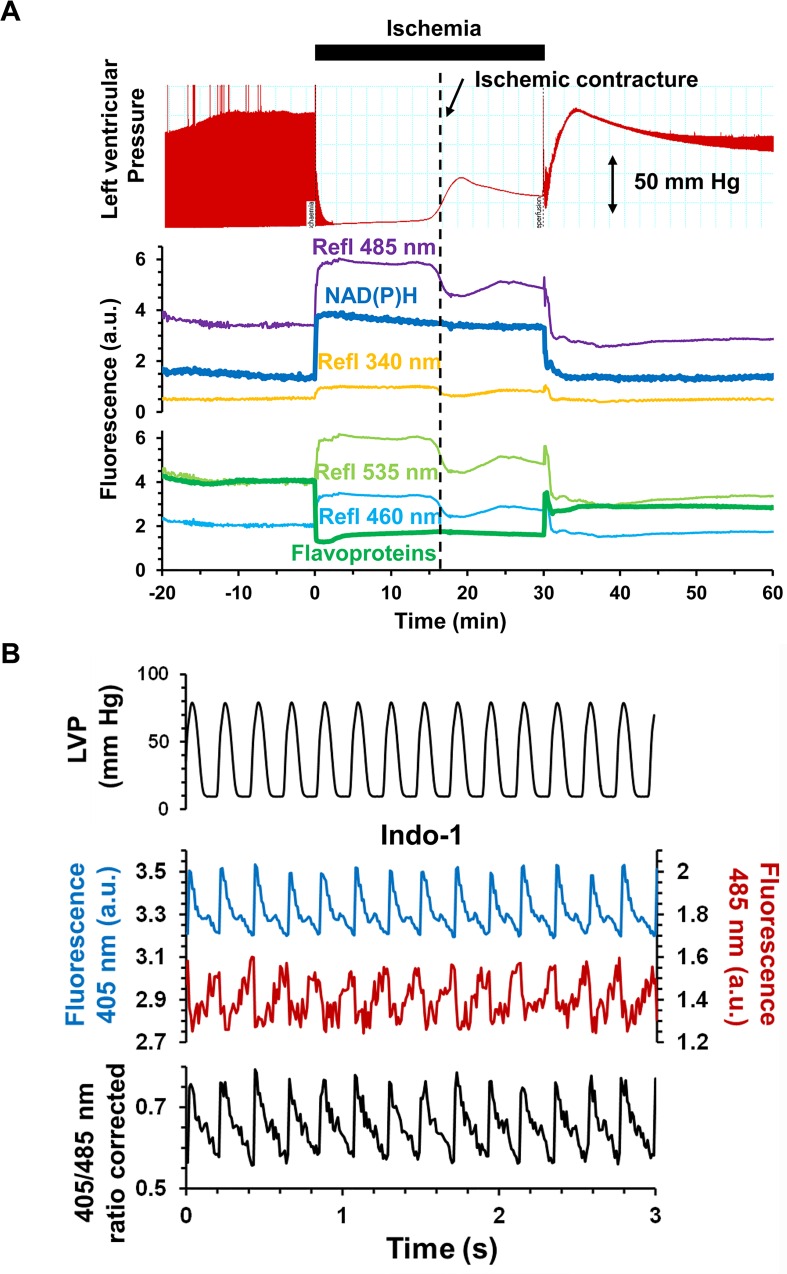
Validation of the bespoke surface fluorescence apparatus. **A,** Time course of NAD(P)H (top) and flavoprotein (bottom) surface fluorescence and the reflectance signals at the corresponding excitation and emission wavelengths together with data for the left ventricular pressure (LVP). **B,** Example of Indo-1 fluorescence transients recorded at 405 nm (in blue) and at 485 nm (in red), and Indo-1 ratio calculated after subtracting background autofluorescence. The top trace shows corresponding LVP data.

We first validated the use of our bespoke surface fluorescence apparatus by monitoring the changes in NAD(P)H and flavoprotein autofluorescence during ischemia and reperfusion (Fig 3A). Upon ischemia, we observed the expected rapid change in reduction state of both NAD(P)H (increase in 340_ex_ / 485_em_ fluorescence) and flavoproteins (decrease in 460_ex_ / 535_em_ fluorescence) and their re-oxidation during reperfusion. An important feature of our optical configuration is that we can measure the reflectance of light at the wavelengths used for both excitation and emission. These signals, included in Fig 3A, will change in response to both the geometry of the heart surface and the internal filtering caused by changes in the absorbance of endogenous chromophores such as myoglobin and cytochromes. However, changes in light scattering dominate because the optic fibre is held 2–3 mm from the surface of the heart to avoid contact artefacts (see Fig 2B). This is apparent in Fig 3A where the reflectance signals at all wavelengths decrease during ischemic contracture (increase in ventricular pressure) which causes the surface geometry of the heart to change. Importantly, contracture caused no significant change in the fluorescence signals implying that changes in surface geometry are not significantly affecting our fluorescence measurements.

We also carried out experiments with rapid data collection to confirm that we could detect beat by beat changes in cytosolic [Ca^2+^], determined using Indo-1 fluorescence (Fig 3B). By using a ratiometric dual emission dye, movement artefacts are minimised because detection of emitted light at both wavelengths is simultaneous, and both are subjected to the same changes in optical geometry caused by the heart beat. Transient changes in the 405/485 nm fluorescence emission ratio of Indo-1 were detected that exactly followed changes in developed pressure monitored using a latex balloon inserted in the left ventricle.

**Fig 4:**
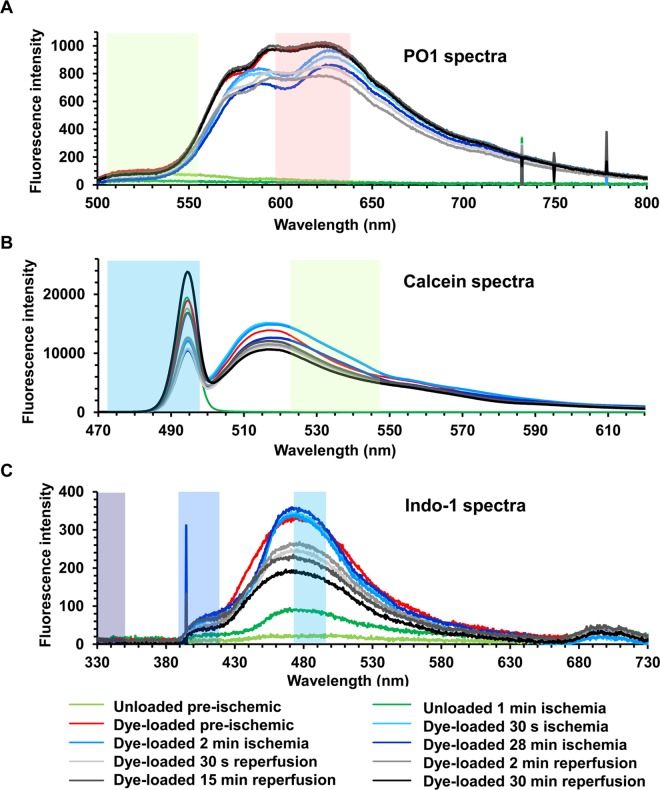
Surface fluorescence spectra of hearts at different stages of dye loading and ischemia-reperfusion protocols. **A**, PO1 perfused hearts; **B**, Calcein loaded hearts; **C**, Indo-1 loaded hearts. The coloured shaded areas indicate the band passes of the filters used for excitation and emission in the filter wheel mode of operation (see S1 Table for details).

As noted above, another potential artefact that might interfere with fluorescent measurements during ischemia and reperfusion is a change in the internal filtering exerted by myoglobin and cytochromes absorbance as they undergo oxygenation / de-oxygenation and oxidation / reduction [17,29]. The most intense absorbance by oxymyoglobin and deoxymyoglobin (absorbance maxima at 420 and 440 nm respectively [30]) occurs outside the range of all dyes used except for Indo-1, and in this case the 405/485 nm emission ratio was employed. This should be relatively insensitive to myoglobin oxygenation state since the 405/485 absorbance ratios of oxymyoglobin and deoxymyoglobin are similar. Furthermore, signals were corrected for autofluorescence by performing parallel experiments with unloaded hearts under identical conditions and instrument settings. In order to assess the extent to which changes in internal filtering might affect fluorescence signals of probes employed in our studies that are excited and emitting at higher wavelengths, we measured surface emission spectra under normoxic conditions and at different times of ischemia and reperfusion. These data are presented in Fig 4 which also includes the wavelength bandwidths of the emission filters we employed. Changes in fluorescence spectra were observed within the first 30 s of ischemia and were rapidly reversed on reperfusion. These changes are likely to be caused by modifications of the internal filtering by myoglobin as it moves between its oxy- and deoxy- state. Indeed, myoglobin is known to be rapidly de-oxygenated upon ischemia (t_0.5_, 6 s) with 90% equilibration reached at 12 s; re-oxygenation at reperfusion occurs at a similar rate [17]. Thus some caution must be employed when interpreting data obtained within the first 10 s or so of ischemia or reperfusion, when the major changes in myoglobin oxygenation state and thus internal filtering occur. However, this is unlikely to account for any differences observed between control and IP hearts whose rates of myoglobin deoxygenation and oxygenation would not be expected to differ greatly. Furthermore,after about 10 s of reperfusion, comparison of fluorescent signals between control and IP hearts should be unaffected by changes in either internal filtering or surface geometry under our experimental conditions.

### ROS measurements

**Fig 5:**
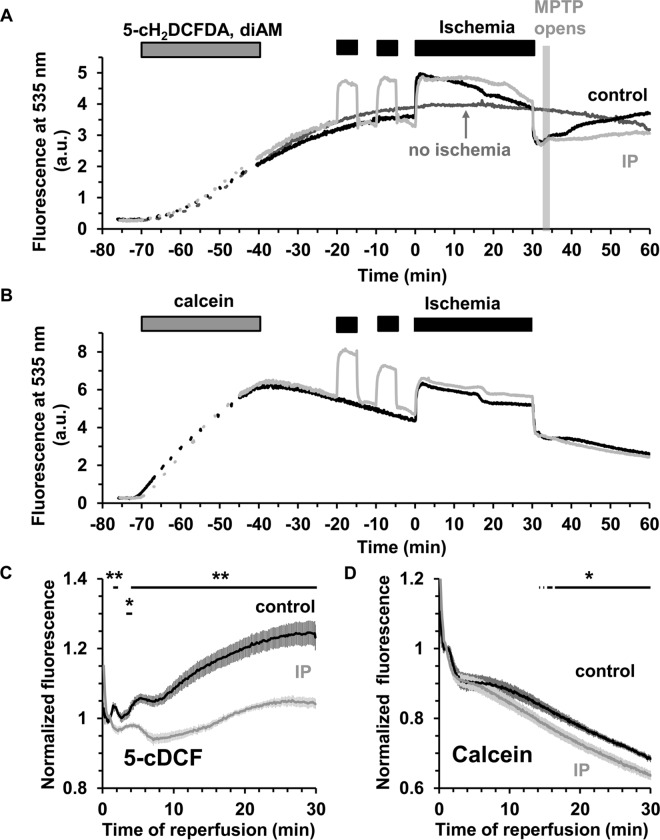
**ROS measurements using 5-cDCF surface fluorescence in control and IP hearts during ischemia / reperfusion A,** Typical traces for 5-cH_2_DCFDA, diAM loaded control and IP hearts. **B,** Parallel data for hearts loaded with the ROS-insensitive dye, calcein-AM. The signal from a non-ischemic 5-cH_2_DCFDA, diAM loaded heart (**A**, dark grey trace) demonstrates good dye retention during the time of the experiment. **C** and **D**, Mean data (± SEM, error bars) of 6 hearts loaded with 5-cH_2_DCFDA, diAM (**C**) or calcein-AM (**D**). Signals were normalized using the mean fluorescence value obtained after 1 min of reperfusion. Note that loss of calcein signal with time is due to dye leakage and occurs even in normoxia. Statistically significant effects of IP are shown by the horizontal lines (** p ≤ 0.01; * p ≤ 0.05). Corresponding infarct sizes are presented in Table 1.

We explored the use of several fluorescent probes that detect different forms of ROS, including dihydroethidium and several dichlorodihydrofluorescein dyes, but found the majority to be unsatisfactory for use with the perfused rat heart for a variety of reasons as outlined in Supporting Information. Nevertheless, we found that 5-carboxy-2', 7'-dichlorodihydrofluorescein diacetate (5-cH_2_DCFDA) in its diAM form consistently loaded into the heart and was quite well retained as illustrated in Fig 5A. However, there is some debate as to the mechanism of 2’,7’-dichlorodihydrofluorescein oxidation in cells and even whether it is suitable to monitor ROS levels [31,32]. Thus we also explored the use of boronate cage reagents have been developed whose fluorescence responds specifically to H_2_O_2_ [33].

### IP attenuates 5-cH_2_DCFDA oxidation during reperfusion

Fig 5A presents typical 5-cH_2_DCFDA fluorescence traces for non-ischemic and ischemic hearts without (control) and with IP. In order to discriminate between fluorescence artefacts caused by changes in internal filtering and real ROS related changes in fluorescence, parallel experiments were performed using hearts loaded with the ROS-insensitive dye, calcein (Fig 5B), whose fluorescence properties closely match those of 5-cDCFDA. Comparison of the 5-cDCFDA and calcein data (Fig 5A vs. 5B) reveals that the very rapid changes of 5-cDCFDA fluorescence at the onset of ischemia and reperfusion are unlikely to be related to ROS production. Rather, they are probably caused by changes in myoglobin absorbance as seen in the fluorescence emission spectra (Fig 4B). However, the slower increase in 5-cDCFDA fluorescence on reperfusion is not seen with calcein and thus would appear to report a real increase in ROS which is largely prevented by IP. Fig 5C presents mean data (± SEM) of 6 separate experiments for control and IP hearts. To correct for slight differences in dye loading between hearts, data were normalized to the fluorescence signal at 1 min of reperfusion. In control hearts, a short transient increase in 5-cDCFDA signal is seen between 1.5 and 2 min followed after 4.2 min by a significant and progressive rise in fluorescence that is not seen in IP hearts (*p* ≤ 0.01). Infarct size data for these hearts after 2 hours reperfusion are reported in Table 1 and confirm that IP was strongly cardioprotective under these conditions (15.3 ± 1.4% *vs*. 60.3 ± 5.4%). The observed increase in 5-cDCFDA fluorescence was not accompanied by a parallel increase in the calcein signal which showed no significant difference between control and IP hearts (Fig 5D). Note that loss of calcein signal with time is due to dye leakage and occurs even in normoxia. The similar dye leakage between control and IP hearts suggests that at the early stage of reperfusion myocyte plasma membranes integrity is similar in control and IP hearts.

**Table 1:** Infarct size and hemodynamic recovery of hearts in all experiments.

		Control					IP				
			Infarct size	RPP	RPP	LVEDP		Infarct size	RPP	RPP	LVEDP
			(% of whole	(mmHg*beat	recovery	(mmHg)		(% of whole	(mmHg*beat	recovery	(mmHg)
Fluorophore	Ins^1^	N	heart)	/min)	(%)		n	heart)	/min)	(%)	
**5cDCF**	+	6	60.3 ± 5.4	22151 ± 2037	10.9 ± 2.1	61.9 ± 4.5	6	15.3 ± 1.4 **	22230 ± 1559	59.5 ± 5 **	35.7 ± 4.5 **
**PO1**	-	11	44.4 ± 3.2	27825 ± 1714	27 ± 4.6	74.7 ± 5.7	9	10.4 ± 0.6 **	27416 ± 1444	102 ± 7.9 **	15.1 ± 2 **
**PO1**	-	17	38.6 ± 3	28269 ± 1164	28.3 ± 3.7	73.4 ± 1	-	-	-	-	-
**PO1 + CsA**	-	16	25.5 ± 1.7 †††	28606 ± 1074	42.2 ± 5.5 †	63.5 ± 3.7	-	-	-	-	-
**MitoPY1**	+	10	47.2 ± 4.7	32162 ± 1353	13.9 ± 2	63.7 ± 2.9	8	33.6 ± 1.9 *§§	37914 ± 772 *§	15.8 ± 3.5§	61.2 ± 2.2
**Mock MitoPY1**	+	6	50.2 ± 6.3	30223 ± 1708	11.9 ± 4.3	74.8 ± 6.3	6	13.0 ± 2.1 **	31827 ± 1703	47.3 ± 7.1 **	46 ± 5.6 **
**Indo-1**	+	15	61.9 ± 2.3	23953 ± 812	11.1 ± 1.5	75.1 ± 3.2	10	13.9 ± 1.7 **	22832 ± 1525	58.2 ± 7.5 **	39.7 ± 3.4 **
**Indo-1 + CsA**	+	10	44.8 ± 3.2 ††	28152 ± 923 ††	24.5 ± 4.7 †	74.7 ± 4.2	-	-	-	-	-

^1^Ins: + or—indicates that 10 nmol/L insulin was present or not in the dye loading solution. Data are taken from the heart perfusions used in the relevant surface fluorescence measurements and are given as means ± SEM for the number of hearts indicated. The absolute RPP and LVEDP values were measured 1 min before the start of global ischaemia or IP. Recovery of RPP after 60 min reperfusion is expressed as a percentage of these preischemic values.

* ≤ p 0.05

** p≤0.01 control vs IP

§ p≤0.05

§§ p≤0.01 MitoPY1 vs Mock MitoPY1

† p≤0.05

†† p≤0.01

††† p≤0.001 control vs control +CsA.

### IP attenuates PO1-detectable hydrogen peroxide production during reperfusion

**Fig 6:**
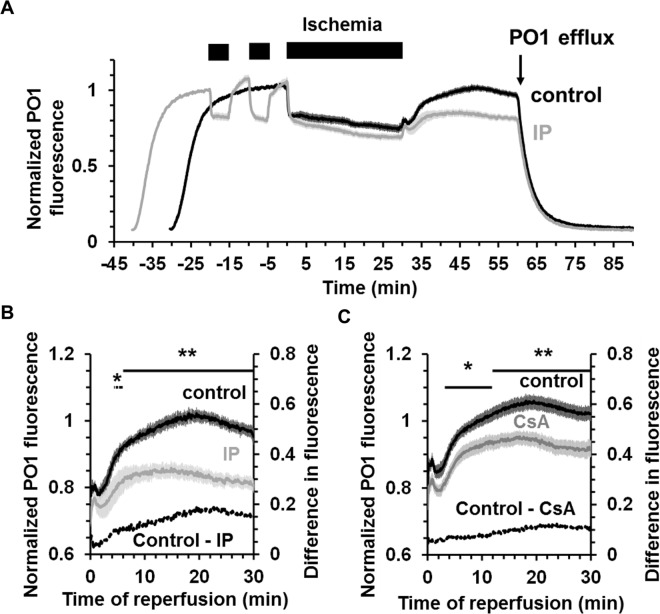
H_2_O_2_ production measured using PO1 fluorescence in control and IP hearts during ischemia / reperfusion. **A**, Averaged fluorescence data for control (black) or IP (grey) hearts perfused with 5 μmol/L PO1. PO1 was present in the perfusion solution from 30 min (Control) or 40 min (IP) before starting global ischemia. PO1 was excluded from the perfusion solution after 30 min of reperfusion. The fluorescence was normalized using the value obtained at -11 min for control and -21 min for IP hearts and then averaged for each group. **B**, Mean data (± SEM as error bars; n = 11 for control and n = 9 for IP hearts) for PO1 fluorescence during the reperfusion period. **C,** Mean data (± SEM as error bars; n = 17 for control and n = 16 for CsA-treated hearts) for PO1 fluorescence during reperfusion of control hearts (black) and CsA-treated hearts (grey). Data were normalized to the fluorescence value at -16 min. Where present, CsA (0.2 mmol/L) was added to the PO1 perfusion solution 15 min before ischemia and during 30 min of reperfusion. Statistically significant effects of IP and CsA are shown by the horizontal lines (* p ≤ 0.05; ** p ≤ 0.01). Corresponding infarct sizes are presented in Table 1.

We first employed PO1 (535_ex_ / 615_em_) to measure global H_2_O_2_ in the heart during ischemia and reperfusion and Fig 6A presents normalised mean data (± SEM) for control and IP hearts. The data show that PO1 was taken up by the perfused heart but not retained upon wash out and therefore must be present in the perfusion medium throughout the experiment. Very rapid decreases in PO1 fluorescent signals were observed upon ischemia with an equally rapid reversal on reperfusion. These changes are similar, but in reverse direction, to those seen for 5-cDCFDA and calcein and might be the result of changes in internal filtering by cytochromes *a* and *a*_*3*_ that have a reduced minus oxidised absorbance maximum of 606 nm in intact mitochondria [34]. This is consistent with the rapid change in fluorescence emission spectra of PO1 shown in Fig 4A. However, we observed an additional, slower increase in PO1 fluorescence that began after about 2–3 min of reperfusion and was greatly inhibited by IP as shown in Fig 6B. This response is very similar to that seen with 5-cDCF (Fig 5C). In a separate set of experiments we investigated the effects of adding 0.2 μmol/L Cyclosporin A (CsA), to inhibit mPTP opening, on the increase in PO1 fluorescence during reperfusion. Fig 6C shows that the increase in signal was diminished, but the effect was much less profound than that induced by IP and only clearly visible after about 8 min as shown in Fig 6D. This is not unexpected since, as reported in Table 1, CsA gave less reduction in infarct size (25.5 ± 1.7% from 38.6 ± 3.0%) than IP (10.4 ± 0.6% from 44.4 ± 3.2%) which is consistent with CsA being less effective than IP at preventing mPTP opening as measured by the mitochondrial deoxyglucose entrapment technique [23].

### Neither MitoPY1 nor aconitase activity detect increased mitochondrial H_2_O_2_ early in reperfusion

**Fig 7:**
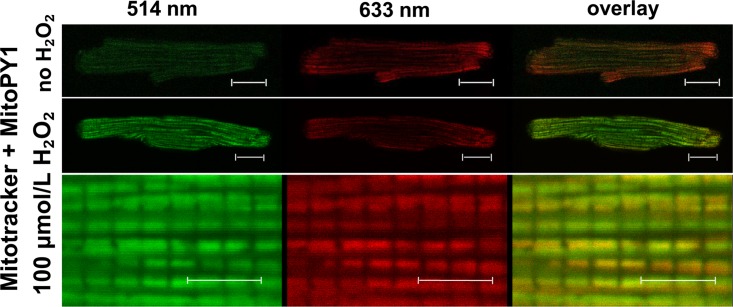
Measurement of mitochondrial H_2_O_2_ in cardiomyocytes using MitoPY1. Confocal imaging of cardiomyoytes loaded with MitoTracker Deep Red and MitoPY1 before and after H₂O₂-challenge. H₂O₂ (100 μmol/L) was added directly to the coverslips and incubated for 10 min before re-imaging again. Scale bars: 20 μm, top panels and 5 μm, bottom panels. As expected, the MitoPY1 signal was low in the basal state but increased substantially upon addition of H_2_O_2_. The high resolution image (bottom panel) reveals individual interfibrillar mitochondria and the overlay of green MitoPY1 signal and red Mitotracker signal (right panels) confirms the dye’s mitochondrial localisation.

**Fig 8:**
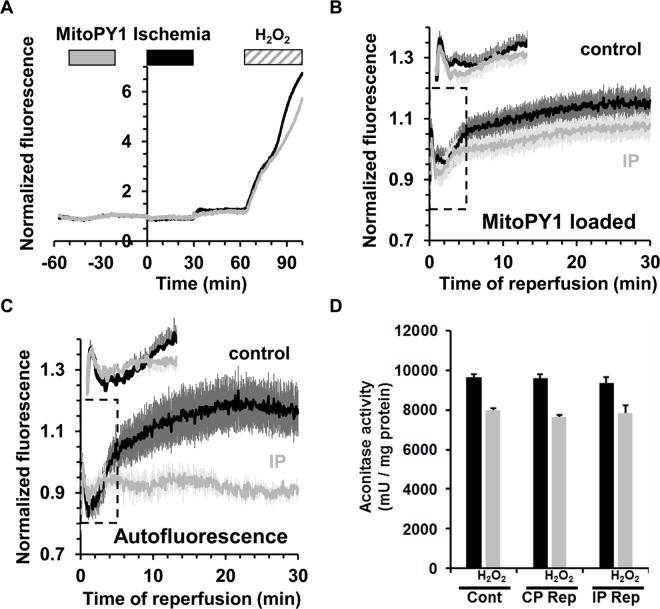
Measurement of mitochondrial H_2_O_2_ following ischemia / reperfusion in the perfused heart using MitoPY1 and aconitase activity. **A**, MitoPY1 fluorescence (535 nm) in control (black) and IP (grey) in Langendorff-perfused hearts subjected to 30 min ischemia + 30 min reperfusion. Fluorescence was normalized to the 1 min pre-iscaemic value. MitoPY1 was successfully loaded into the hearts as shown by the increase in fluorescence upon addition of H_2_O_2_ to the perfusion medium. **B,** Mean 535 nm fluorescence (± SEM as error bars) was normalized to the average value between 20.5 and 21.5 min obtained on reperfusion after 30 min global ischemia in control (black, n = 10) and IP hearts (grey, n = 8). **C**, Corresponding autofluorescence data for hearts subject to a mock loading protocol (n = 6 in both control and IP hearts). Corresponding infarct sizes are presented in Table 1. **D**, Aconitase activity in mitochondria isolated from normoxic control hearts (Cont) and both control (CP Rep) and ischemic preconditioned (IP Rep) hearts subjected to 30 min ischemia and 90 s reperfusion as described in S1 Fig The grey bars confirm the reduction in aconitase activity following treatment of the mitochondrial extract with 200 μmol/L H_2_O_2_. Data are given as means ± SEM (error bars) of 4 hearts in each group.

In order to measure changes in mitochondrial matrix ROS during reperfusion we first employed MitoPY1, a boronate-cage H_2_O_2_-specific fluorescent probe that is targeted to mitochondria using the positively charged triphenylphosphonium group [35]. We established that MitoPY1 is correctly targeted to mitochondria in isolated cardiac myocytes and confirmed that it responded to H_2_O_2_ (Fig 7). We then loaded MitoPY1 into the Langendorff-perfused hearts and demonstrated that it responded to added H_2_O_2_ in this setting (Fig 8A). However, unlike for PO1 and 5-cDCFDA, we were unable to demonstrate any significant changes between control and IP hearts in the small fluorescence increase upon reperfusion (Fig 8B). Although there appeared to be a trend towards a lower signal in the IP hearts, the data of Fig 8C suggest this is probably caused by changes in autofluorescence rather than MitoPY1 itself. It should also be noted that the MitoPY1-loaded IP hearts showed a significantly greater infarct size than unloaded hearts (Table 1) suggesting that MitoPY1 loading might attenuate the effects of IP by some unidentified mechanism. Overall, we conclude that the small changes in the MitoPY1 signal observed were more likely to represents changes in flavoprotein autofluorescence (485 nm excitation) and that any increase in matrix H_2_O_2_ upon reperfusion is below our limit of detection.

Further evidence against any major increases in mitochondrial ROS occurring during early reperfusion was obtained by measuring aconitase activity in mitochondria isolated after 90 s of reperfusion. No significant differences in activity were detected between pre-ischemic and 90 s reperfused samples, whereas treatment of the mitochondrial extracts (at 0°C) with 200 μmol/L H_2_O_2_ did reduce enzyme activity by about 20% (Fig 8D). Significant decreases (~50%) in aconitase activity on reperfusion have been detected by others [20], but the earliest time point measured was after 15 min of reperfusion when our PO1 and 5-cDCF data also detect increases in ROS.

### The effects of IP on tissue succinate and autofluorescence are inconsistent with complex 1 mediated generation of matrix superoxide in early reperfusion

**Fig 9:**
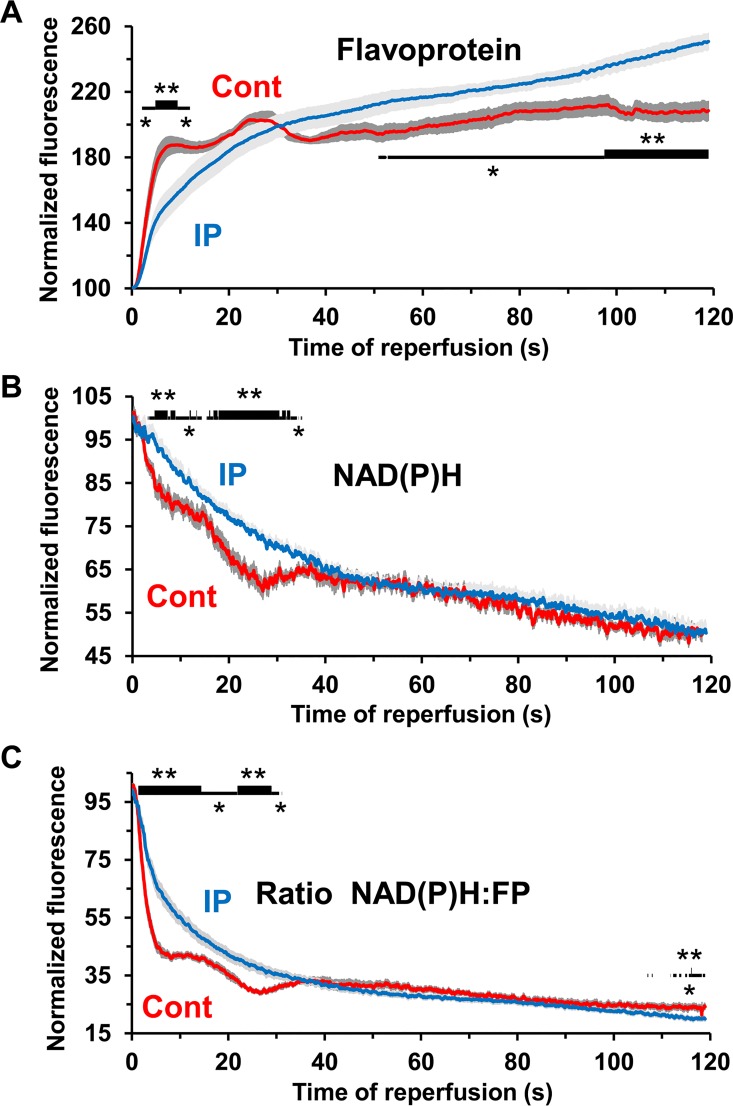
Rapid re-oxidation of NAD(P)H and flavoproteins upon reperfusion of control and IP hearts following 30 min global ischemia. Data are presented as means ± SEM (error bars) of 8 control and 9 IP hearts (* p<0.05; ** p<0.01). S2 Table presents mean data for the corresponding values at 1 min before ischemia and at 2, 10 and 30 min of reperfusion, while a full representative trace for each parameter is given in S2 Fig

Chouchani *et al*. have proposed that superoxide production occurs early in reperfusion from the matrix face of Complex 1 which they suggest is maintained in a highly reduced state by reverse electron flow mediated by the oxidation of succinate that accumulates during ischemia [20,36]. In order to investigate this possibility we have monitored the redox state of NAD(P)H and flavoproteins over the first two minutes of reperfusion using continuous acquisition at 10 Hz. In Fig 9 mean data (± SEM), normalised to the end ischemic value, are presented for control (n = 8) and IP hearts (n = 9). Since the fluorescence of NAD(P)H decreases on re-oxidation (Panel A), while that of flavoproteins increases (Panel B), we also calculated the ratio (Panel C) since this will reduce any errors caused by motion artefacts. In control hearts the redox state of flavoproteins and NAD(P)H returned to pre-ischemic levels with a characteristic and very reproducible multiphase pattern that included a very rapid initial oxidation (t_0.5_ < 10 s). In IP hearts re-oxidation showed only a single phase and was significantly slower than for control hearts (t_0.5_ ~15 s). We have also measured the levels of succinate in hearts and found pre-ischemic values were below our limit of detection (~0.2 nmol/mg dry weight (dw)) but increased to 4.82 ± 0.47 nmol/mg dw (n = 6) after 30 min ischemia as described by Chouchani *et al* [20]. However, a similar increase in succinate to 3.91± 0.38 nmol/mg dw (n = 6) was also observed in IP hearts subject to 30 min ischemia arguing against reverse electron from succinate being the source of ROS at complex 1 that is modulated by IP.

### Measurement of intracellular [Ca^2+^] during ischemia / reperfusion using Indo-1

**Fig 10:**
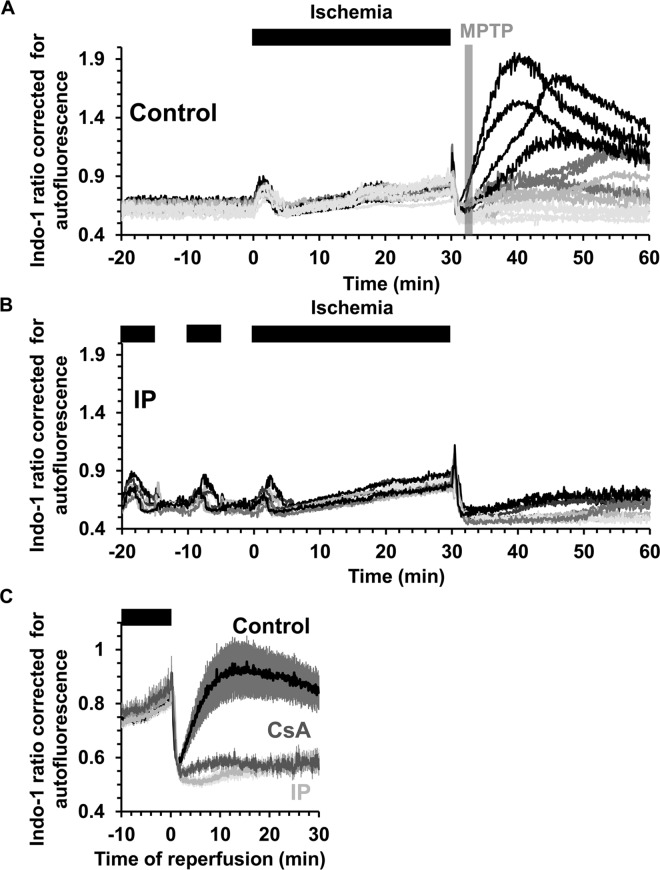
Measurement of [Ca^2+^] using Indo-1 in control and IP hearts during ischemia / reperfusion. Indo-1 fluorescence ratio corrected for autofluorescence was monitored in control (**A**) and IP (**B**) hearts undergoing 30 min global ischemia and reperfusion. **C**, Mean data (± SEM as error bars) for control (black, n = 15), IP (light grey, n = 10) and CsA-treated hearts (dark grey, n = 10) during the last 10 min of ischemia and 30 min of reperfusion. In CsA-treated hearts, 0.2 μmol/L CsA was present in the perfusion medium for 10 min before ischemia and for 30 min of reperfusion (n = 10). Corresponding infarct sizes are presented in Table 1.

**Fig 11:**
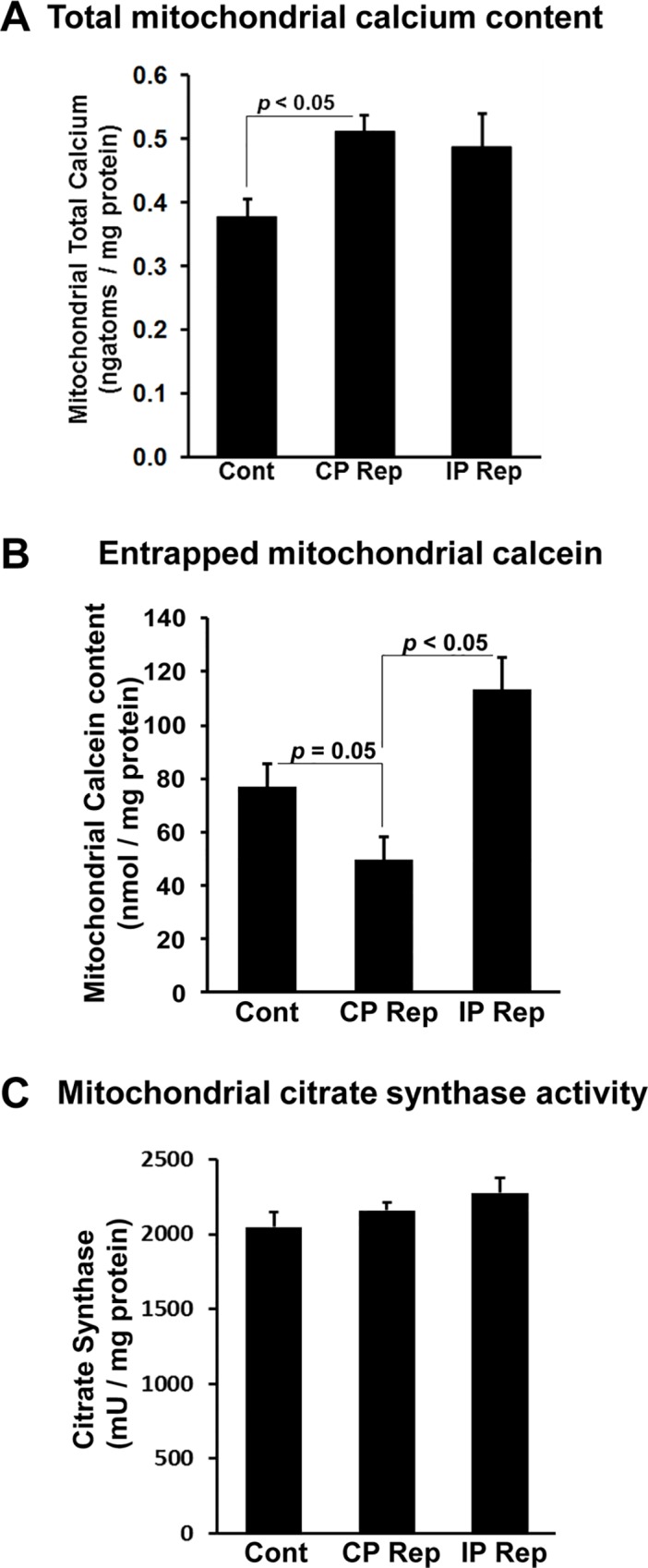
Measurement of total calcium content, entrapped calcein and citrate synthase activity in mitochondria isolated from control and IP hearts after ischemia / reperfusion. Mitochondria were isolated from normoxic control hearts (Cont) and both control (CP Rep) and IP (IP Rep) hearts subject to 30 min ischemia and 90 s reperfusion as described in S1 Fig. Data are given as means ± SEM (error bars) of 4 hearts in each group.

We monitored [Ca^2+^]_i_ using Indo-1 and it is important to note that some of this dye loads into mitochondria [26] and thus its response may reflect changes in mitochondrial as well as cytosolic [Ca^2+^]. Fig 10 shows individual traces for changes in the Indo-1 ratio corrected for background autofluorescence in control (panel A) and IP (panel B) hearts. In both cases, during the first 2–3 min of ischemia the Indo-1 ratio rises and then decreases again before rising continuously until the end of 30 min of ischemia. Since the binding of Ca^2+^ to Indo-1 is pH sensitive [37], the decrease in pH during ischemia could initially attenuate or reverse any increase caused by an increase in cytosolic [Ca^2+^]. Indeed, this may account for the initial rise and fall in signal. Nevertheless, the continuous rise between 5–30 min of ischemia does reflect a real and progressive increase in [Ca^2+^], but importantly there is no significant difference between control and IP hearts. However, a major difference in cytosolic [Ca^2+^] between control and IP hearts is seen upon reperfusion as highlighted in Fig 10C where mean data (± SEM) for 15 control hearts and 10 IP hearts are presented. In both cases the signal initially drops rapidly, most likely reflecting a decrease in cytosolic [Ca^2+^] as it is removed from the cytosol by export from the cells and uptake into the SR and re-energized mitochondria. Indeed, in a separate set of experiments in which we isolated mitochondria at 90 s of reperfusion under conditions that maintain their calcium content, we found that the total matrix calcium (ngatoms/mg protein and means ± SEM) increased from 0.380 ± 0.028 in preischemic hearts to 0.501 ± 0.022 and 0.486 ± 0.054 in control and IP hearts respectively (Fig 11A).

As reperfusion continued the Indo-1 signal stayed low in IP hearts (infarct size 13.9 ± 1.7%) with only a gradual increase upon reperfusion, whereas in control hearts (infarct size 61.9 ± 2.3%), the signal began to rise rapidly after about 2 min, although with substantial variation between hearts as seen in Fig 10A. This rise probably occurs after mPTP opening since we have shown previously, using the deoxyglucose entrapment technique, that mPTP opening begins within 2 min of reperfusion and is largely prevented by IP [23]. However, in order to confirm this to be the case under our current experimental conditions, we isolated mitochondria from hearts loaded with calcein (S1 Fig). The calcein-AM that enters mitochondria *in situ* will be trapped within them following AM ester hydrolysis, but will be lost following mPTP opening and isolation of mitochondria. At 90 s of reperfusion mitochondria isolated from control I/R hearts showed significantly less entrapped calcein than mitochondria from pre-ischemic hearts (Fig 11B). This confirms that some mPTP opening had occurred at this time, whereas for IP hearts this was not the case, consistent with inhibition of mPTP opening. These data support our conclusion that the onset of the rise in cytosolic [Ca^2+^] during reperfusion follows rather than precedes mPTP opening. Additional evidence for this is provided by the data of Fig 10D which shows that the presence of 0.2 μmol/L CsA prior to ischemia and during reperfusion to inhibit mPTP opening, also largely prevented this [Ca^2+^] rise while reducing infarct size from 61.9 ± 2.3 to 44.8 ± 3.2%.

### Opening of the mPTP in isolated heart mitochondria induces ROS formation

**Fig 12:**
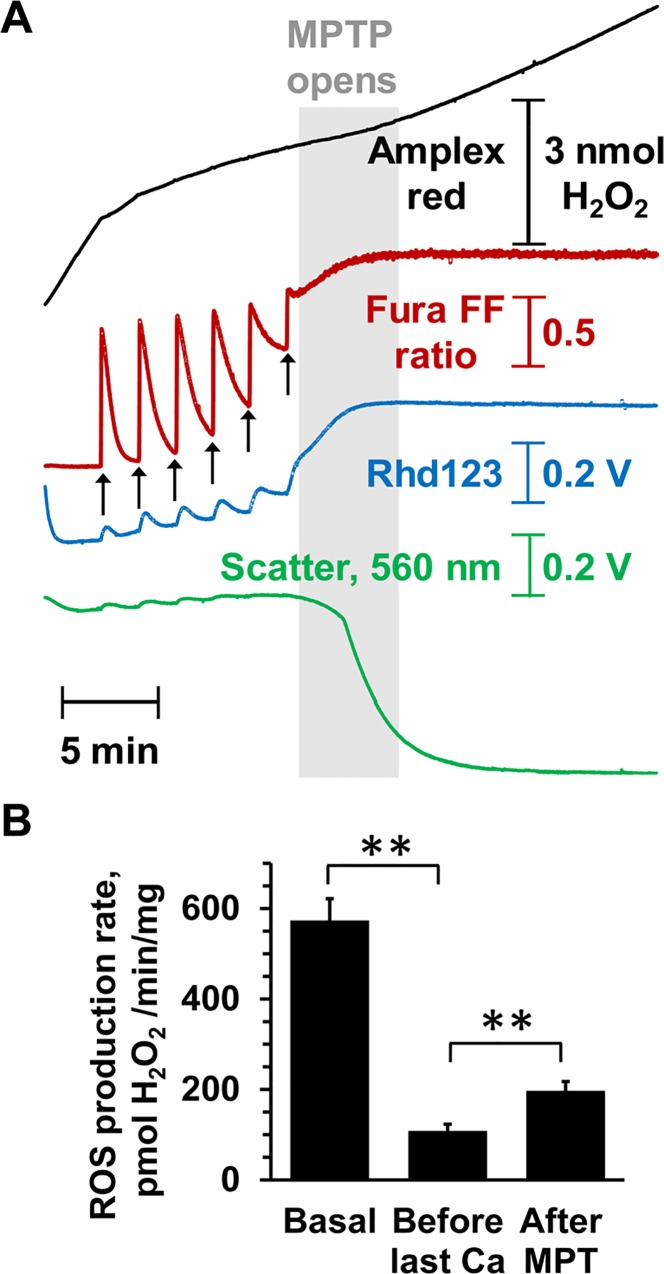
ROS production by isolated mitochondria before and after MPTP opening. **A,** Effects of sequential additions of Ca^2+^ (100 mmol/L) to isolated rat heart mitochondria incubated with 5 mmol/L L-glutamate + 2 mmol/L L-malate and 5 mmol/L succinate (GMS) on mPTP opening measured as the loss of ΔΨ (Rhd-123 fluorescence increase) or accumulated Ca^2+^ (Fura-FF fluorescence increase) and decrease in LS. ROS production was determined using Amplex Red in a parallel experiment on the same batch of mitochondria. **B,** Mean rates of ROS production (± SEM of 6 different mitochondrial preparations) before Ca^2+^ addition, after the penultimate Ca^2+^ addition and following pore opening. ** p ≤ 0.01.

Our data suggest that the major rise in ROS and [Ca^2+^] observed after the first few minutes of reperfusion is a consequence of mPTP opening. In Fig 12 we present data to confirm that opening of the mPTP does greatly enhance ROS production and release accumulated [Ca^2+^], consistent with this prediction. Isolated heart mitochondria were energised with glutamate + malate and succinate (GMS) to mimic the substrate availability *in situ* [28], and simultaneous measurements made of light scattering (LS), mitochondrial membrane potential (ΔΨ_m_) and extramitochondrial [Ca^2+^]. Parallel experiments monitored H_2_O_2_ emission with Amplex Red and LS. Fig 12A shows that sequential additions of 100 μmol/L Ca^2+^ were rapidly taken up by the mitochondria with a progressive reduction in ΔΨ_m_ and consequently ROS production rate until the mPTP opened (rapid and total loss of ΔΨ_m_, release of accumulated Ca^2+^ and a large LS decrease). At this point, ROS production greatly increased again. These data are quantified in Fig 12B.

## Discussion

Although it is widely accepted that increases in mitochondrial matrix [Ca^2+^] and ROS trigger mPTP opening early in reperfusion [3,4], this has not been adequately demonstrated in the beating perfused heart subject to ischemia and reperfusion. The bespoke surface fluorescence equipment we describe in this paper, together with a robust experimental approach has allowed us to address many of the substantial challenges associated with such measurements. Our data lead us to the conclusion that reduction in ROS and [Ca^2+^] early in reperfusion may not be the primary mechanism by which IP inhibits mPTP opening.

### Validation and limitations of surface fluorescent techniques to measure ROS and Ca^2+^ during ischemia / reperfusion of the beating heart

Although major changes in surface reflectance signals were detected in response to ischemic contracture, they were not accompanied by significant changes in NAD(P)H and flavoprotein autofluorescence (Fig 3A). This confirms the reliability of our fluorescence measurements even in the face of the significant changes in optical geometry occurring at the onset of both ischemia and reperfusion. Concerns over fluorescence artefacts caused by changes in internal filtering by myoglobin and cytochromes during ischemia and reperfusion were addressed by measuring the fluorescence emission spectrum from the surface of the heart in pre-ischemic hearts and at different times of ischemia and reperfusion (Fig 4). The data show that although there are potential artefacts caused by such changes, they are likely to affect only the first few seconds of ischemia and reperfusion and most importantly to be similar in control and IP hearts. Furthermore, we employed two different ROS-fluorescent dyes with distinct spectral properties and ROS specificity: 5-cH_2_DCFDA (loaded as diAM), whose oxidation occurs in response to oxidative stress but which may not directly monitor ROS [31,32], and the H_2_O_2_ specific probe, PO1 [33]. Reassuringly, we obtained similar data with both. In the case of 5-cH_2_DCFDA we also performed parallel experiments with the ROS-insensitive dye calcein, which has similar fluorescent properties to 5-cDCFDA, and confirmed that it did not show a similar increase in fluorescence on reperfusion. Thus we are confident that the time course of ROS change we observe with both dyes accurately reflects global changes in ROS.

Measurements of [Ca^2+^] using the 405/485 nm fluorescence emission ratio of Indo-1 are relatively insensitive to changes in the internal filtering effects caused by changes in myoglobin oxygenation or cytochrome oxidation state (Fig 4). In addition, we performed parallel experiments in the absence of Indo-1 (at the same instrument settings) to correct for changes in autofluorescence. We have not attempted to correct for the influence of pH on the response of Indo-1 to [Ca^2+^] since we are interested in detecting major differences between control and IP hearts rather than absolute [Ca^2+^] values, and differences in pH are unlikely to explain the ability of IP to prevent the large increases in [Ca^2+^] observed later in reperfusion.

We recognise that an additional limitation of our surface fluorescence measurements is their restriction to measuring ROS and [Ca^2+^] changes in the epicardium, but this is true of any optical technique that uses emission and excitation wavelengths close to 500nm. It is also possible that the magnitude and time courses of fluorescence changes observed in the epicardium may not exactly mirror those in more internal layers of the ventricle wall. However, our measurements of mitochondrial aconitase activity and calcium content suggest that any such differences would not significantly affect our conclusions since mitochondria were isolated from the whole ventricle and these show effects entirely consistent with those obtained by the fluorescence measurements. Thus at 90 s of reperfusion, no change in aconitase activity was detected (Fig 8D) in agreement with our inability to detect significant ROS increases with 5-cH_2_DCFDA (Fig 5C), PO1 (Fig 6B) or MitoPY1 (Fig 8B). Nor did IP attenuate the increase in mitochondrial Ca^2+^ content on reperfusion (Fig 11A) consistent with no change in the Indo-1 fluorescence data at this time point (Fig 10C).

### The role of reverse electron flow in superoxide production at complex 1

Chouchani *et al*. [20] have proposed that superoxide production occurs early in reperfusion from the matrix face of Complex 1 which they suggest is maintained in a highly reduced state by reverse electron flow mediated by the oxidation of the succinate that accumulates during ischemia [20,36]. However, our measurements of the redox state of NAD(P)H and flavoproteins in the first two minutes of reperfusion (Fig 9) revealed that they were both very rapidly re-oxidised (t_0.5_ < 15 s) and that this re-oxidation was slower in IP than control hearts. This would appear to be inconsistent with IP reducing superoxide production during early reperfusion. However, the data are consistent with IP enhancing inhibition of the mitochondrial ATP synthase by its inhibitor IF1 during ischemia, as has been observed in some studies (see [38]) since this could lead to slower activation of oxidative phosphorylation and respiration on reperfusion. Furthermore, the accumulation of succinate during 30 min ischemia was not prevented by IP (3.91± 0.38 nmol/mg dw (n = 6) compared to 4.82 ± 0.47 nmol/mg dw (n = 6) in controls). Thus if an increase in ROS is occurring early in reperfusion to stimulate mPTP opening our data suggest it is not a result of reverse electon flow from succinate leading to increased superoxide production at Complex 1. We next address whether such an increase in matrix ROS early in reperfusion does occur prior to initial mPTP opening.

### Detectable ROS increases start at 2–3 min of reperfusion, after mPTP opening, and are inhibited by IP and CsA

A wealth of published data, including our own [3], led us to anticipate that we would observe a significant increase in ROS immediately on reperfusion that would act as a trigger for mPTP opening, and that this would be reduced by IP. However, neither 5-cDCFDA nor PO1 signals began to rise consistently until 2–3 min of reperfusion (Figs 5C and 6C) which is after mPTP opening occurs, as revealed in early experiments by increased entrapment of 2-deoxyglucose [2] and in the present experiments by release of entrapped calcein (Fig 11B). It is possible that 5-cDCFDA and PO1 are insufficiently sensitive to detect any small early increases in mitochondrial matrix ROS that might be responsible for triggering mPTP opening. However, our measurements of NAD(P)H and flavoproteins autofluorescence during the first phase of reperfusion do not suffer these limitations. As noted above, these data reveal that NAD(P)H and flavoproteins are oxidised very rapidly (t_0.5_ < 10 s) and so would be unlikely to generate significant ROS after the first few seconds of reperfusion. Equally important is the observation that the rate of oxidation of NAD(P)H and flavoproteins is slower in IP hearts than in control hearts. If complex 1 were producing a rapid burst of ROS responsible for triggering mPTP opening our data would predict greater ROS production in the mitochondria of IP hearts and thous would be expected to enhance rather than impair mPTP opening. Furthermore, we were unable to detect any significant increase in mitochondrial H_2_O_2_ using the targeted probe MitoPY1 (Fig 8B) or aconitase activity (Fig 8D) after 90 s of reperfusion. Again, we recognize that these techniques suffer limitations. Thus loading with MitoPY1 appeared to have additional “off-target” effects that modulate the infarct size (Table 1), most notably increasing it in IP hearts from 13.2 ± 2.1% to 33.6 ± 1.9%. Nor can we completely rule out the possibility that the sensitivity and kinetics of the reaction of MitoPY1 and aconitase with H_2_O_2_ are sufficient to detect any small increases in matrix H_2_O_2_ early in reperfusion that might trigger mPTP opening. A compounding factor for MitoPY1 is that small changes in its signal during the first 10–20 seconds of reperfusion could be masked by major changes in internal filtering caused by myoglobin re-oxygenation that occurs over this time period [17] as well as by rapid changes in autofluorescence (Fig 8C). Nevertheless, even taking these limitations into account, our data clearly show that any changes in mitochondrial matrix ROS in early reperfusion must be very small compared with those observed later in reperfusion. It should be noted that although Chouchani *et al*. [20] did detect an increase in H_2_O_2_ using the mitochondrial-targeted boronate cage reagent MitoB and aconitase activity, this was measured at 15 min of reperfusion, again after mPTP opening has peaked [2] and when our own data confirm that a significant increase in ROS has occurred. However, it is also important to note that our data do not rule out the possibility that there is a rapid burst of superoxide production in the early stages of reperfusion which is immediately removed by endogenous defense mechanisms such as superoxide dismutase working in combination with glutathione peroxidase and glutathione reductase. The subsequent rises in ROS we observe later in reperfusion might reflect the exhaustion of these defence mechanisms.

Overall, our data suggest that any increases in mitochondrial matrix H_2_O_2_ early in reperfusion are too small to be detected by the techniques we employed and thus are unlikely to mediate the initial IP-inhibitable opening of the mPTP. This may explain why our earlier studies with the mitochondria-targeted superoxide scavenger, MitoQ, failed to demonstrate cardioprotection in the Langendorff-perfused heart [39]. It should be noted that earlier data showing protection from I/R injury of the heart by MitoQ was obtained in a whole animal model in which rats were treated for 14 days with mitoQ in their drinking water before induction of myocardial ischemia [40]. Thus the observed cardioprotective effects of the drug in these experiments could be secondary to its longer term effects and not directly via mitochondrial ROS scavenging in the heart. Rather, our data suggest that the observed increase in ROS on reperfusion detected with both 5-cDCFDA (Fig 5C) and PO1 (Fig 6B) occurs after mPTP opening and is attenuated by IP and CsA as a secondary response to their inhibition of pore opening. It should be noted that superoxide production occurring after mPTP opening, whether in the matrix or the intermembrane space, will be detected by extramitochondrial ROS probes such as 5-cDCFDA and PO1 that we employed.

The reduction in ROS caused by IP is considerably greater than that by CsA (Fig 6C) and this matches the relative ability of IP and CsA to prevent mPTP opening on reperfusion as determined with the 2-deoxyglucose entrapment protocol [23]. The data we present in Fig 12 confirm that increased ROS production does occur following mPTP opening in isolated heart mitochondria as has previously been reported [41]. It is probably the result of cytochrome *c* and NADPH loss from the mitochondria, both of which are important for ROS scavenging [21,41]. Indeed it has been proposed that such mPTP-mediated ROS production drives progressive mPTP opening in adjacent mitochondria and cells leading to an expanding wave of necrotic cell death as reperfusion continues [3,41]. However, the presence of ROS scavengers during reperfusion has proved to be relatively ineffective at preventing IR in either the clinical setting or with *in vivo* and *ex vivo* animal models of IR [42,43]. A better understanding of the mechanisms leading to ROS production later during the reperfusion phase may help the developement of drugs that inhibit this process more effectively, although a role of ROS in cardioprotective signalling pathways [43,44] may work against this strategy. Furthermore, it is also possible that mPTP-induced ROS production is less important in causing a progressive cascade of mPTP opening than the dysregulation of [Ca^2+^] discussed below.

### IP inhibits the large increases in [Ca^2+^] during reperfusion that occur after mPTP opening

Another major trigger for mPTP opening is matrix [Ca^2+^]. As predicted, we did detect an increase in Indo-1 ratio during ischemia, consistent with a rise in cytosolic and mitochondrial [Ca^2+^], and this was followed by a return to basal at the start of reperfusion (Fig 10A), most likely reflecting cytosolic calcium efflux from the cardiomyocytes and / or uptake into mitochondria and other intracellular stores. Similar data were obtained with 20 min ischemia by Hassinen’s group using Fura-2, but this dual wavelength excitation indicator (340/380_ex_, >515_em_) requires much greater corrections for autofluorescence than Indo-1 [30,45]. We were also able to demonstrate that mitochondria isolated after 90 s of reperfusion contained more calcium than pre-ischemic hearts consistent with calcium being an important trigger for mPTP opening. However, our data revealed that the increase in [Ca^2+^] during ischemia in IP hearts was similar to that in control hearts (Fig 10A and 10B), as was the total calcium content of mitochondria isolated at 90 s of reperfusion (Fig 11A). Thus it is unlikely that IP modulates mPTP opening on reperfusion by attenuating [Ca^2+^] within the mitochondrial matrix. Nevertheless, after 2–3 min of reperfusion, control hearts did show a profound and progressive increase in Indo-1 ratio which was not observed in IP hearts (Fig 10C). This large rise in [Ca^2+^] later in reperfusion, like that of ROS, is likely to be secondary to mPTP opening and probably reflects energy compromise in cells following mPTP opening with subsequent impairment of ionic homeostasis. Indeed we show that treatment of hearts with CsA to inhibit mPTP opening also largely prevented the observed increase in [Ca^2+^] on reperfusion (Fig 10C). Similar observations have been made by others using an isolated myocyte model of ischemia / reperfusion [46]. The dysregulation of intracellular [Ca^2+^] following initial mPTP opening may act in conjunction with the rise in ROS to cause additional mPTP opening in adjacent “unopened” mitochondria and lead to a progressive wave of cell death as discussed above. This may explain the waves of [Ca^2+^] and ROS preceding mPTP opening that have been observed by confocal microscopy in the intact contraction-inhibited heart subject to hypoxia and reoxygenation [18].

It is important to stress that our data do not rule out a role for mitochondrial Ca^2+^ accumulation at the end of ischemia and during early reperfusion in mPTP opening. Such increases in matrix [Ca^2+^] have been demonstrated previously in both cardiac myocytes [47] and perfused hearts [48] and confirmed here (Fig 11A). Indeed, hearts of mice with an adult cardiomyocyte-specific deletion of the mitochondrial calcium uniporter (MCU) have recently been shown to exhibit reduced IR injury [49,50] confirming earlier data obtained with the MCU inhibitors ruthenium red and Ru360 [51,52]. However, what our data do imply is that inhibition of mPTP opening by IP is unlikely to be secondary to decreased matrix calcium overload, but rather by decreased sensitivity to matrix [Ca^2+^].

### Implications for cardioprotection by therapeutic interventions

Prevention of mPTP opening at reperfusion is widely regarded as a promising target for cardioprotection [3,8]. In the context of cardiac surgery, preconditioning (ischemic and pharmacological) is feasible but this is not the case for treating a coronary thrombosis by percutaneous coronary intervention (PCI–also called angioplasty). This may explain why clinical trials failed to show a significant cardioprotective effect of CsA given intravenously prior to the treatment of coronary thrombosis with PCI [53]. However, under these conditions, ischemic post-conditioning may be effective. Here the relevant coronary arteries are subject to short transient cycles of blood flow and occlusion for the first few minutes of reperfusion [54]. Although the mechanisms underlying ischemic post-conditioning are not fully elucidated, it has been shown to be associated with inhibition of mPTP opening. This may be partially explained by maintenance of an acid pH (inhibitory for mPTP opening) for longer during the early stages of reperfusion, but other signalling pathways are also thought to be involved [44,55]. There are no published data to show that reduced ROS production during the early stages of reperfusion post-conditioning is the mechanism by which it inhibits mPTP. However, this is not unexpected in view of our inability to detect increases in ROS early in reperfusion. Although we have not confirmed it experimentally, we would suggest that no matter how post-conditioning inhibits the first phase of mPTP opening, it would prevent secondary ROS production and dysregulation of [Ca^2+^] with less subsequent mPTP opening and thus reduced infarct formation. Indeed, our data lead us to predict that any protocl employed during reperfusion that reduces disturbances in ROS and calcium during the later phase of the reperfusion should be a good strategy to limit further mPTP opening and thus provide cardioprotection.

## Conclusions

**Fig 13:**
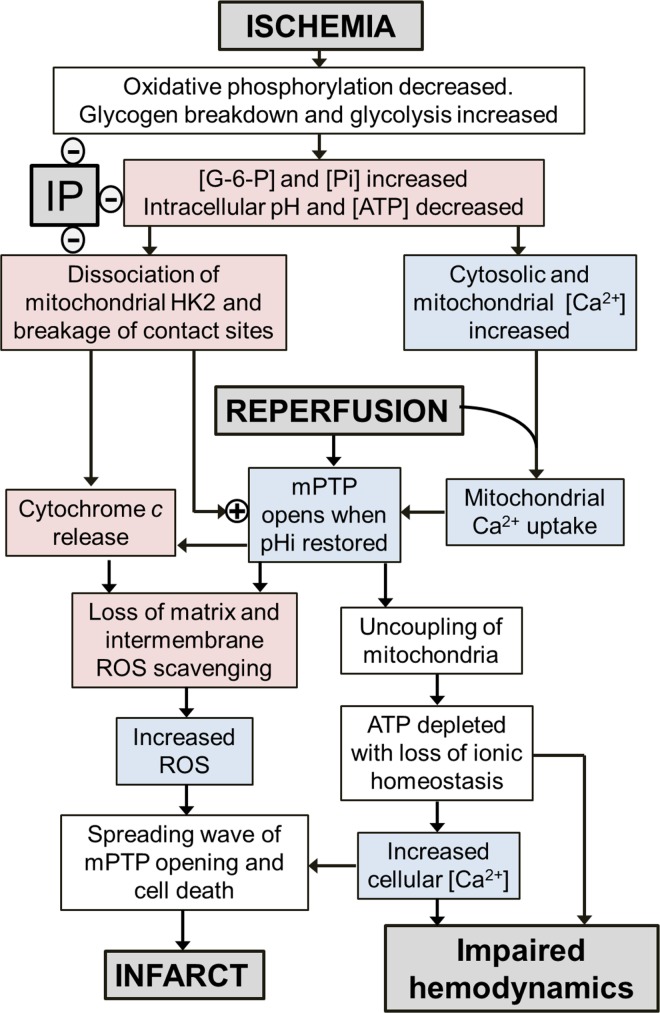
Schematic to show proposed sequence of events relating ischemia and reperfusion to mPTP opening and increases in ROS and [Ca^2+^]. Events confirmed in the present paper are shown in lilac and those previously reported in [21] are shown in mauve.

Taken together, our data suggest that any changes in [Ca^2+^] or ROS early in reperfusion that might trigger mPTP opening are small compared with those later in reperfusion, and not attenuated by IP. If this is the case, IP would have to work through alternative mechanisms to attenuate initial mPTP opening. One such established mechanism involves hexokinase 2 (HK2) whose binding to mitochondria is associated with resistance to mPTP opening [22,56,57] and also with stabilisation of contact sites between the inner and outer membrane whose breakage may enhance cytochrome *c* release [56,58]. HK2 is known to dissociate from heart mitochondria during reperfusion and this is prevented by IP [21,22,56,57]. Indeed, there is a very strong inverse correlation between the amount of HK2 remaining bound to mitochondria at the end of ischemia and the infarct size after 120 min of reperfusion [21]. We propose that it may be these changes to the mitochondria together with the elevated [Ca^2+^] present at the end of ischemia and during early reperfusion that sensitise mitochondria to mPTP opening. As reperfusion continues, the intracellular pH returns to control values (>7) from end-ischemic values (< 6.5) that inhibit mPTP opening. As a result, mPTP opening now occurs and leads to the observed secondary increase in ROS release. In addition, through impaired energy metabolism and consequent dysregulation of calcium homeostasis, a large rise in [Ca^2+^] occurs. These secondary changes in [Ca^2+^] and ROS, will then trigger mPTP opening in adjacent mitochondria and neighbouring cells leading to a spreading wave of mPTP opening and increasing infarct size as reperfusion continues. IP, by preventing the HK2 loss and contact site destabilization during ischemia, will prevent these secondary increases in [Ca^2+^] and ROS and thus attenuate the resulting cell death and infarct development. This is illustrated schematically in Fig 13. We have suggested previously how a wide range of cardioprotective strategies and signalling pathways may exert their effects through this common pathway [56].

## Supporting Information

S1 Fig Perfusion protocol and hemodynamic parameters for heart perfusions used to determine mitochondrial calcium content, calcein loading and aconitase activity. Panel **A:** Protocol for the three groups of hearts studied: a normoxic control group (Cont), a control reperfused group (CP Rep) and ischemic preconditioned reperfused group (IP Rep). Panels **B** and **C:** LVEDP during ischemia and reperfusion respectively. Panel **D**: Myocardial oxygen consumption. Data are given as Means ± SEM (error bars) of 4 hearts in each group.
(TIF)

S2 Fig Autofluorescence of NAD(P)H and flavoproteins during I/R protocol. Panel **A**: Representative traces of flavoprotein fluorescence of IP and control heart. Panel **B**: Representative traces of NAD(P)H fluorescence. Panel **C**: NAD(P)H to flavoprotein ratio.
(TIF)

S1 File Supplementary Methods. (DOCX)

S2 File Supplementary Results. (DOCX)

S1 Table Details of filters used and photomultiplier voltage used. (DOCX)

S2 Table Mean values of autofluorescence data before ischemia and during reperfusion normalised to the value at the end of 30 min ischemia. These data are taken from the same heart perfusions as for Fig 2B where the detailed time courses of the authofluorescence changes during the first 2 min of reperfusion are presented.
(DOCX)
