# Supplementary figures and images for: Real-Time Fluorescence Measurements of ROS and [Ca2+] in Ischemic / Reperfused Rat Hearts: Detectable Increases Occur only after Mitochondrial Pore Opening and Are Attenuated by Ischemic Preconditioning
Source: PLoS One. 2016 Dec 1;11(12):e0167300. doi: 10.1371/journal.pone.0167300 (PMC5131916; doi:10.1371/journal.pone.0167300)

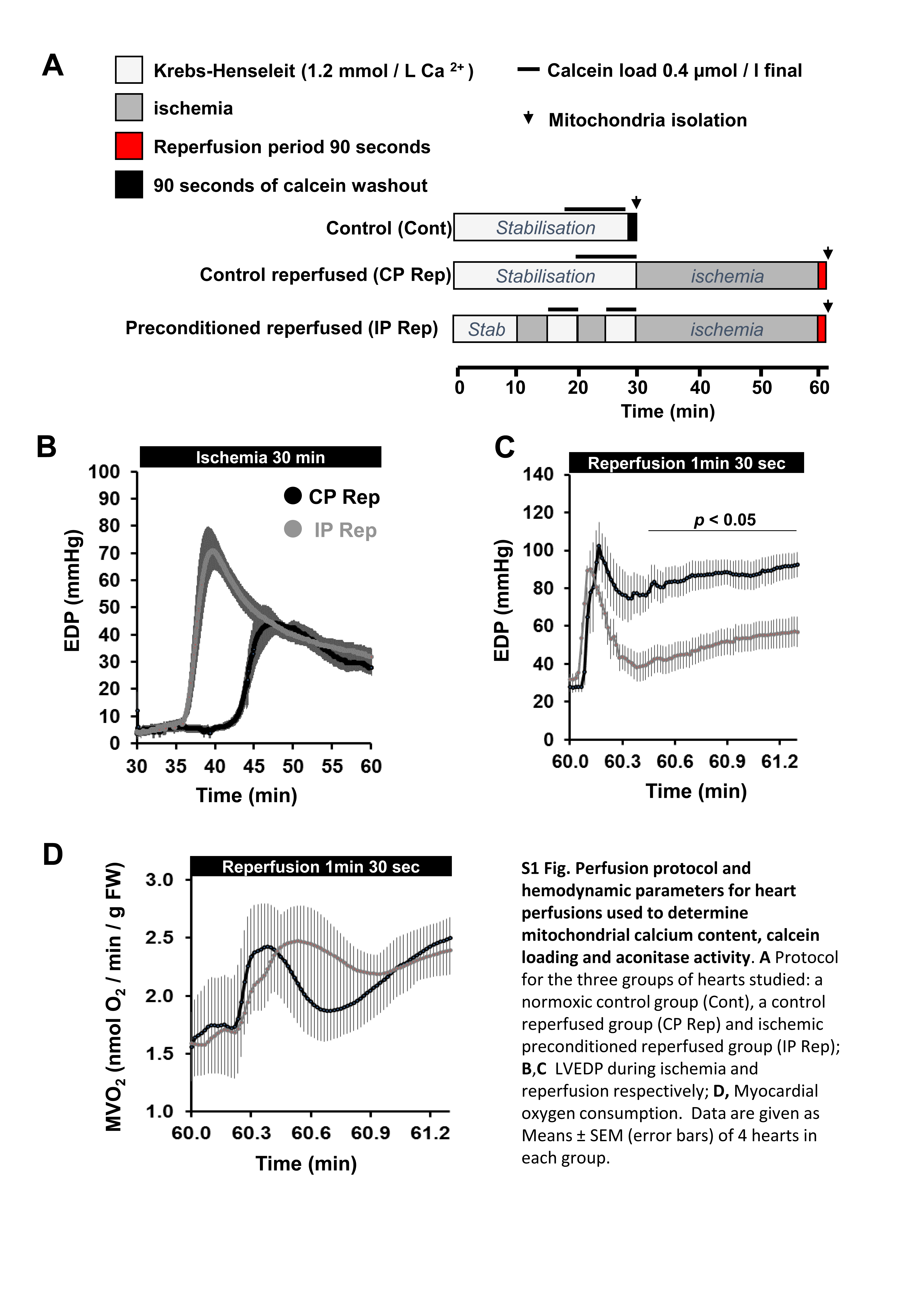

Supplement: S1 Fig — Panel A: Protocol for the three groups of hearts studied: a normoxic control group (Cont), a control reperfused group (CP Rep) and ischemic preconditioned reperfused group (IP Rep). Panels B and C: LVEDP during ischemia and reperfusion respectively. Panel D: Myocardial oxygen consumption. Data are given as Means ± SEM (error bars) of 4 hearts in each group. (TIF) [file pone.0167300.s001.tif]

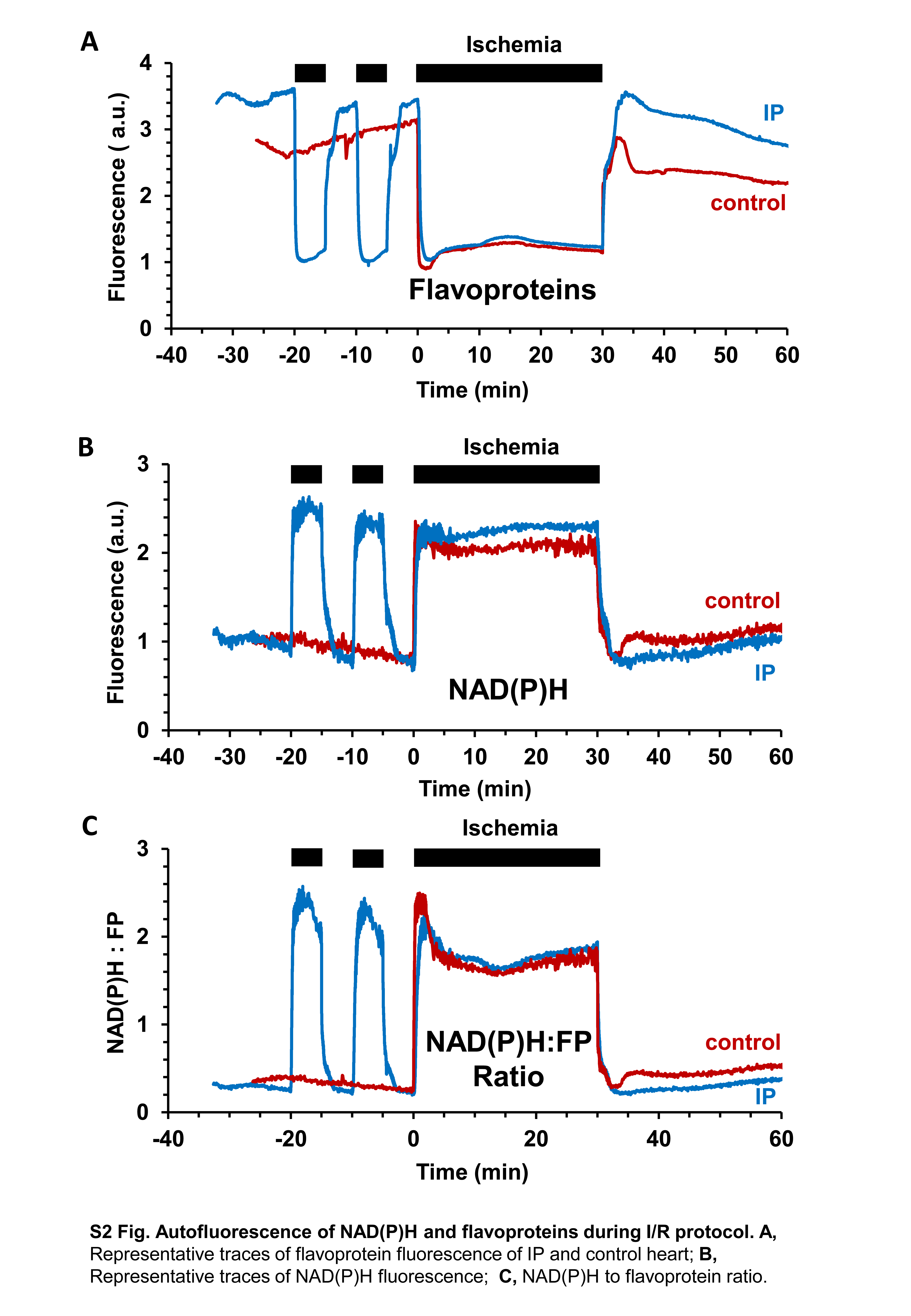

Supplement: S2 Fig — Panel A: Representative traces of flavoprotein fluorescence of IP and control heart. Panel B: Representative traces of NAD(P)H fluorescence. Panel C: NAD(P)H to flavoprotein ratio. (TIF) [file pone.0167300.s002.tif]
