# Supplementary material for: Real-Time Fluorescence Measurements of ROS and [Ca2+] in Ischemic / Reperfused Rat Hearts: Detectable Increases Occur only after Mitochondrial Pore Opening and Are Attenuated by Ischemic Preconditioning
Source: PLoS One. 2016 Dec 1;11(12):e0167300. doi: 10.1371/journal.pone.0167300 (PMC5131916; doi:10.1371/journal.pone.0167300)
Supplement: S1 File — (DOCX) [file pone.0167300.s003.docx]

**Real-Time Fluorescence Measurements of ROS and [Ca^2+^] in Ischemic / Reperfused Rat Hearts: Detectable Increases Occur only after Mitochondrial Pore Opening and are Attenuated by Ischemic Preconditioning**

Tatyana N Andrienko, Philippe Pasdois Andreas Rossbach, and Andrew P Halestrap

**Supplementary Methods**

Additional details of the techniques used for heart perfusion and fluorescence measurements are provided below.

**Langendorff heart perfusion with measurement of hemodynamic function and infarct size**

This study was carried out in strict accordance with the UK Animals (Scientific Procedures) Act 1986 and the guidelines from the Directive 2010/63/EU of the European Parliament. The study and all protocols employed were also approved by the local ethical committees of the University of Bristol (UB/09/012) and University of Bordeaux (Authorization number A33-318-2). Male Wistar rats (250–300 g) were killed either by stunning and cervical dislocation (Bristol) or anesthetized by 3% isoflurane, heparinized and euthanized by a lethal IP injection of 130 mg/kg pentobarbital (Bordeaux). All efforts were made to minimize suffering.

Hearts were rapidly excised, rinsed in ice-cold Krebs–Henseleit Buffer (KHB) containing (in mmol/L) NaCl 118, NaHCO_3_ 25, KCl 4.8, KH_2_PO_4_ 1.2, MgSO_4_ 1.2, glucose 11 and CaCl_2_ 1.2, gassed with 95% O_2_/5% CO_2_ at 37°C (pH 7.4) and cannulated. The base of the pulmonary artery was cut to assist drainage. Heart perfusions were performed in Langendorff mode with KHB at constant flow 12 mL/min essentially as described previously [1].

For measurements of surface fluorescence (carried out in Bristol), perfusion was performed within the light-proof box of the surface fluorescence apparatus described below. Perfusion protocols are summarized in Fig. 1. A water-filled latex balloon was inserted into the left ventricle for continuous monitoring of heart rate (HR) and left ventricular pressure (LVP) using PowerLab with LabChart v7.0 software (AD Instruments). The volume of the balloon was set to give an initial left ventricular end diastolic pressure (LVEDP) of 5–10 mmHg. Left ventricular developed pressure (LVDP) was calculated as the difference between left ventricular systolic pressure (LVSP) and LVEDP (LVDP = LVSP − LVEDP). The rate-pressure product (RPP), calculated as RPP = LVDP × HR, was used as an index of cardiac work. Ischemic preconditioning (IP) was elicited by two cycles of 5 min of global ischemia (stopping the aortic inflow) interspersed with 5 min reperfusion prior to 30 min global (index) ischemia. The heart was maintained in the water-jacketed humidified Plexiglas perfusion chamber at 37°C throughout the perfusion (Fig. 2A). After 120 min reperfusion hearts were analysed for infarct size by perfusing for 2 min at 10 mL/min with 1% (w/v) of 2’,3’,5’-triphenyltetrazolium chloride (TTC) solution prepared in Phosphate-Buffered Saline (PBS). After 5 min incubation at 37°C hearts were sliced into 5-6 slices and incubated overnight in 4% (w/v) formaldehyde solution in PBS at 4°C. Both sides of each slice were photographed and then were dried for 24h and weighted. The surfaces of the necrotic and total area of each side for each slice were determined by planimetry (AlphaEase v5.5), and because global ischemia was used, infarct size was expressed as a percentage of the total cross-sectional area of the heart.

For experiments involving measurement of myocardial oxygen consumption, mitochondrial calcium content, calcein loading and aconitase activity (carried out in Bordeaux) Langendorff heart perfusions were performed under identical conditions but using a different custom built apparatus that allowed simultaneous monitoring of oxygen consumption and hemodynamic function. Hearts were perfused in a constant flow mode (12 ml/min) according to the protocol illustrated in Panel A of S1 Fig. which also contains data for the LVEDP (Panels B and C) and oxygen consumption (Panel D) of these hearts. Three groups of hearts were studied; a normoxic control group (Cont), a control reperfused group (CP Rep) and an ischemic preconditioned reperfused group (IP Rep). Left ventricular pressure was recorded by an EMKA-IOX2 data acquisition system (EMKA Technologies, Falls Church, VA).

**Myocardial Oxygen Consumption (MVO_2_)**

The tip of a 6-cm-long polyethylene drain was placed in the pulmonary artery to collect coronary effluent for myocardial oxygen consumption (MVO_2_) measurements. Coronary effluent was anaerobically collected at 1.5 mL/min by a pump connected to an oxymeter (Strathkelvin Instruments). PO_2_ was continuously monitored throughout the experiments and recorded at 100 Hz by an EMKA-IOX2 data acquisition system (EMKA Technologies, Falls Church, VA). Perfusate PO_2_ was measured immediately above the heart at the beginning and at the end of each experiment, and MVO_2_ (µmol O_2_/min/g fw) was determined as shown below:

$${MVO}_{2}=(Perfusate O_{2}-effluent O_{2})\times\left( \frac{Coronary output}{Heart weight} \right)$$

Perfusate O_2_ content and effluent O_2_ content were expressed in μmol/mL; coronary output was expressed in mL/min; heart weight was expressed as g of fresh weight (gfw). Consequently, MVO_2_ was expressed in µmol O_2_/min/g fw.

**Whole heart surface fluorescence measurements.**

Epicardial fluorescence was monitored using a spinning wheel fluorimeter. This was designed by two of us (PP and APH) with the advice of Dr Martyn Reynolds of Cairn Research Ltd (Faversham, Kent, ME13 8UP) who custom-built the acquisition equipment. The modified perfusion apparatus that accommodates the optic fiber from the fluorimeter was designed and built in house (by PP) and contained within a light-proof box. The equipment is shown in Fig. 2A,B and illustrated schematically in Fig. 2C. Excitation light (Xenon Arc) is passed through a 6-position filter wheel and led to the heart surface through one branch of a bifurcated quartz fiber-optic cable (diameter 3.18 mm) which was placed at 2-3 mm distance from the left ventricular wall (see Fig. 2B). The apex of the heart is placed within a small plastic funnel to gently restrict the heart movement on the cannula. The fiber-optic holder is heated with a coil to 40^°^C to avoid condensation on the fiber-optic surface. The other branch of the fiber-optic collects the reflected and emission light and splits the light beam (10% for reflectance and 90% for fluorescence) through 2 additional 6-position filter wheels onto two photomultipliers. All three filter wheels rotate in synchrony, usually at 10 Hz but with 50 Hz used when measuring calcium transients. All filters are interference filters (bandwidths between 20-50 nm) for the wavelengths indicated in Fig. 2 while details of the filters used are given in S1 Table which also provides the photomultiplier voltage settings used. Photomultiplier signals were gated electronically to provide outputs corresponding to each wavelength pair and these are integrated and displayed using the Cairn Data Acquisition Engine software. To enable the use of the dual-emission fluorescent dye, Indo-1, to measure [Ca^2+^]_i_, an additional dichroic filter is located prior to the fluorescence emission filter wheel and directs fluorescent light <440 nm to a third photomultiplier. This dichroic filter can be replaced by a mirror and scanning monochromator Mini-spectrometer TM-VIS/NIR (Hamamatsu Photonics K.K., Hamamatsu-City, Japan) to determine the emission spectra of fluorescent light from the heart surface. To avoid tissue light damage and photobleaching of dyes during long perfusion protocols, a shutter was deployed in front of the excitation light, opening for 1 s every 5 or 10 s and the data collected during this 1 s were averaged during the analysis to yield 1 data point per second.

**Fluorescence measurements of ROS and Ca^2+^**

The protocols for loading the heart with various fluorescent dyes (5-carboxy-2’,7’-dichlorodihydrofluorescein diacetate, di(acetoxymethyl ester) (5-cH_2_DCFDA, diAM), calcein-AM, Peroxy Orange 1 (PO1), Mitochondria Peroxy Yellow 1 (MitoPY1) and Indo-1-AM used to monitor ROS and [Ca^2+^] are shown in Fig. 1. Protocols presented in Figs. 1A and 1B required the presence of 10 nmol/L insulin in the KHB perfusion medium during dye loading to maintain glycogen levels over the extended pre-ischemic perfusion period. In its absence glycogen became depleted and this gave significant cardioprotection [2,3]. In all experiments employing acetoxymethyl ester (AM dyes), the KHB was also supplemented with 0.1 mmol/L probenecid to limit dye leakage and Pluronic F-127 was added to the stock solutions of the dyes to aid their solubilisation (see below). Dye loading started about 7-8 min after the heart cannulation and was preceded by measurement of background fluorescence for 3-5 min.

For measurement of ROS we used several dyes whose loading protocols differed as noted below. Hearts were loaded with 5-carboxy-2’,7’-dichlorodihydrofluorescein diacetate, di(acetoxymethyl ester) (5-cH_2_DCFDA, diAM) using a syringe pump containing 120 µmol/L 5-cH_2_DCFDA, diAM, Pluronic F-127 (0.24% w/v) and 240 nmol/L insulin in KHB. The pump introduced its contents at 0.5 mL/min into the oxygenated KHB perfusing the heart to give final concentrations of 5-cH_2_DCFDA, diAM, pluronic acid and insulin of 5 µmol/L, 0.01% and 10 nmol/L respectively. After 30 min loading the dye was allowed to de-esterify further for 20 min prior to the IP protocol or 40 min for control hearts (*i.e.* 40 min before ischemia in both cases). Calcein was used as a ROS-insensitive dye with similar fluorescent properties to 5-cDCF and was loaded using calcein-AM at a final concentration of 0.4 µmol/L. Two other ROS-sensitive dyes were also employed which are selectively oxidised by H_2_O_2_, Mitochondria Peroxy-Yellow 1 (MitoPY1, Ex 485 nm, Em 535 nm) and Peroxy-Orange 1 (PO1, Ex 535 nm, Em 615 nm). The loading procedure for MitoPY1 was essentially the same as for 5-cH_2_DCFDA, diAM with the final concentration of dye and pluronic acid being 3 μmol/L and 0.006% (w/v) respectively. Parallel experiments were performed using the same loading protocol but without dye to measure autofluorescence at the same wavelength pair and instruments settings. For PO1, which unlike 5-cH_2_DCFDA, diAM and MitoPY1 is not retained within the cell after its removal from the perfusion, the dye was present at 5 μmol/L throughout the entire experiment together with Pluronic F-127 (0.01% w/v) but no probenecid. Insulin was also unnecessary because the loading procedure was shorter.

For measurements of intracellular [Ca^2+^] hearts were loaded with Indo-1 by perfusing for 45 min in recirculating mode (with perfusate re- oxygenation) using KHB containing 3 µmol/L Indo-1 AM, 10 nmol/L insulin, 0.1 mmol/L probenecid and 0.01% (w/v) Pluronic F-127. This was followed by normal perfusion (without Indo-1) for 20 min to complete dye de-esterification prior to either the IP protocol or 20 min further normoxic perfusion (control) and then 30 min index ischemia in control hearts and reperfusion. Excitation was at 340 nm and fluorescence simultaneously recorded at 405 nm and 485 nm. Background fluorescent signals (using identical settings) were collected from hearts subject to mock loading without Indo-1 and subtracted before calculating the F405/F485 ratio. When recording [Ca^2+^] transients over short periods, the shutter was not employed and the filter wheels were rotated at 50 Hz with data acquisition at 100 Hz.

**References**

1. Javadov SA, Clarke S, Das M, Griffiths EJ, Lim KHH, Halestrap AP. Ischaemic preconditioning inhibits opening of mitochondrial permeability transition pores in the reperfused rat heart. J Physiol. 2003; 549: 513-524.

2. Cross HR, Opie LH, Radda GK, Clarke K. Is a high glycogen content beneficial or detrimental to the ischemic rat heart? A controversy resolved. Circ Res. 1996; 78: 482-491.

3. Pasdois P, Parker JE, Halestrap AP. Extent of Mitochondrial Hexokinase II Dissociation During Ischemia Correlates With Mitochondrial Cytochrome c Release, Reactive Oxygen Species Production, and Infarct Size on Reperfusion. J Am Heart Assoc. 2012; 2: e005645.
