# Supplementary material for: Real-Time Fluorescence Measurements of ROS and [Ca2+] in Ischemic / Reperfused Rat Hearts: Detectable Increases Occur only after Mitochondrial Pore Opening and Are Attenuated by Ischemic Preconditioning
Source: PLoS One. 2016 Dec 1;11(12):e0167300. doi: 10.1371/journal.pone.0167300 (PMC5131916; doi:10.1371/journal.pone.0167300)
Supplement: S2 File — (DOCX) [file pone.0167300.s004.docx]

**Real-Time Fluorescence Measurements of ROS and [Ca^2+^] in Ischemic / Reperfused Rat Hearts: Detectable Increases Occur only after Mitochondrial Pore Opening and are Attenuated by Ischemic Preconditioning**

Tatyana N Andrienko, Philippe Pasdois Andreas Rossbach, and Andrew P Halestrap

**Supplementary Results**

**Choice of ROS probes**

We explored the use of several fluorescent probes that detect different forms of ROS but found the majority to be unsatisfactory for use with the perfused rat heart. We initially employed dihydroethidium, but in agreement with others [1,2] we found that this dye exhibited fluorescence signals unrelated to superoxide production. We next investigated the use of different dichlorodihydrofluorescein dyes that are oxidised by ROS to the fluorescent dichlorofluorescin form (485_ex_/535_em_), although the form of ROS detected remain uncertain [3,4]. We found that 2',7'-dichlorodihydrofluorescein diacetate was poorly retained by the heart after loading, and leakage was too great during our long ischemia / reperfusion protocol to allow its use. The newer derivative 5-(and 6-)chloromethyl-2',7'-dichlorodihydrofluorescein diacetate, acetyl ester is commonly used for isolated cardiomyocytes, but the amount of dye required for loading the perfused heart would makes its use prohibitively expensive. We found that 6-carboxy-2',7'-dichlorodihydrofluorescein diacetate did not accumulate in the heart during Langendorff perfusion, while its di-acetoxymethyl ester (diAM) loaded better but gave inconsistent data in our hands. However, we discovered that 5-carboxy-2', 7'-dichlorodihydrofluorescein diacetate (5-cH_2_DCFDA) in its diAM form loaded consistently as illustrated in Fig. 5A.

**References**

1. Zhao H, Joseph J, Fales HM *et al*. Detection and characterization of the product of hydroethidine and intracellular superoxide by HPLC and limitations of fluorescence. Proc Natl Acad Sci U S A. 2005; 102: 5727-5732.

2. Näpänkangasa JP, Liimatta EV, Joensuuc P, Bergmann U , Ylitalo K, Hassinen IE. Superoxide production during ischemia−reperfusion in the perfused rat heart: A comparison of two methods of measurement. J Mol Cell Cardiol. 2012; 53:906-915.

3. Karlsson M, Kurz T, Brunk UT, Nilsson SE, Frennesson CI. What does the commonly used DCF test for oxidative stress really show? Biochem J. 2010; 428: 183-190.

4. Dikalov SI, Harrison DG. Methods for detection of mitochondrial and cellular reactive oxygen species. Antioxid Redox Signal. 2014; 20: 372-382.
