# Supplementary material for: Real-Time Fluorescence Measurements of ROS and [Ca2+] in Ischemic / Reperfused Rat Hearts: Detectable Increases Occur only after Mitochondrial Pore Opening and Are Attenuated by Ischemic Preconditioning
Source: PLoS One. 2016 Dec 1;11(12):e0167300. doi: 10.1371/journal.pone.0167300 (PMC5131916; doi:10.1371/journal.pone.0167300)
Supplement: S1 Table — (DOCX) [file pone.0167300.s005.docx]

**S1 Table. Details of filters used and photomultiplier voltage used**

|  | Excitation Filter Wavelength and Bandpass | | | Fluorophores | High Voltage (Volts) | | |
| --- | --- | --- | --- | --- | --- | --- | --- |
| Position | Excitation  (1) | Reflectance  (2) | Fluorescence  (3) |  | PMT1 | PMT2* | PMT 3 |
| 1 | D460/20x | D460/20x | HQ535/50x | Flavoproteins | 480 | - | 300 |
| 2 | HQ615/45x | HQ615/45x | Blank | Not used | - | - | - |
| 3 | D485/25x | D485/25x | D535/25m | 5cDCF  calcein  MitoPY1  mock MitoPY1 | 290  290  450  450 | -  -  -  - | 290  290  290  290 |
| 4 | D340v2x | D340v2x | D485/25m | NAD(P)H  Indo-1 (50Hz/10Hz) | 480  500/430 | 500/430 | 300  400/350 |
| 5 | D440/20x | D440/20x | D535/25m | Not used | - | - | - |
| 6 | HQ535/50x | HQ535/50x | HQ615/45x | PO1 | 350 | - | 300 |

*The filter in front of PMT2 (Indo-1 emission) was D405/30m.
