# Supplementary material for: Real-Time Fluorescence Measurements of ROS and [Ca2+] in Ischemic / Reperfused Rat Hearts: Detectable Increases Occur only after Mitochondrial Pore Opening and Are Attenuated by Ischemic Preconditioning
Source: PLoS One. 2016 Dec 1;11(12):e0167300. doi: 10.1371/journal.pone.0167300 (PMC5131916; doi:10.1371/journal.pone.0167300)
Supplement: S2 Table — These data are taken from the same heart perfusions as for Fig 2B where the detailed time courses of the authofluorescence changes during the first 2 min of reperfusion are presented. (DOCX) [file pone.0167300.s006.docx]

**Table S2 Mean values of autofluorescence data before ischemia and during reperfusion normalised to the value at the end of 30 min ischemia.** These data are taken from the same heart perfusions as for Fig. 2B where the detailed time courses of the authofluorescence changes during the first 2 min of reperfusion are presented.

| **Time** |  | **1 min before ischemia** | **2 min of reperfusion** | **10 min of reperfusion** | **30 min of reperfusion** |
| --- | --- | --- | --- | --- | --- |
| **Flavoproteins** | **Control** | 273.5 ± 10 | 208.5 ± 6.1 | 207.7 ± 3.9 | 197.7 ± 6.3 |
|  | **IP** | 288.8 ± 8.7 | 250.8 ± 5.3 | 273.6 ± 6.2 | 247.6 ± 0 |
| **NAD(P)H** | **Control** | 42.5 ± 2.9 | 49.9 ±1.8 | 52.5 ± 1.6 | 58.7 ± 2.9 |
|  | **IP** | 35.5 ± 1.4 | 51 ± 2.7 | 43.2 ± 1.5 | 47.3 ± 1.4 |
| **NAD(P)H : Flavoproteins** | **Control** | 16 ± 1.7 | 24.2 ± 1.2 | 25.30 ± 0.72 | 30.1 ± 1.9 |
|  | **IP** | 12.45 ± 0.85 | 19.91 ± 0.95 | 15.85 ± 0.74 | 19.24 ± 0.97 |
